# The critical role of amygdala subnuclei in nociceptive and depressive-like behaviors in peripheral neuropathy

**DOI:** 10.1038/s41598-018-31962-w

**Published:** 2018-09-11

**Authors:** Midiã D. J. Seno, Danielle V. Assis, Flávia Gouveia, Geiza F. Antunes, Mayra Kuroki, Caroline C. Oliveira, Lennon C. T. Santos, Rosana L. Pagano, Raquel C. R. Martinez

**Affiliations:** Laboratory of Neuroscience, Hospital Sirio-Libanes, Sao Paulo, SP Brazil

## Abstract

The amygdala is an important component of the limbic system that participates in the control of the pain response and modulates the affective-motivational aspect of pain. Neuropathic pain is a serious public health problem and has a strong affective-motivational component that makes it difficult to treat. The central (CeA), basolateral (BLA) and lateral (LA) nuclei of the amygdala are involved in the processing and regulation of chronic pain. However, the roles of these nuclei in the maintenance of neuropathic pain, anxiety and depression remain unclear. Thus, the main objective of this study was to investigate the role of amygdala subnuclei in the modulation of neuropathic pain, including the affective-motivational axis, in an experimental model of peripheral neuropathy. The specific goals were as follows: (1) To evaluate the nociceptive responses and the patterns of activation of the CeA, BLA and LA in neuropathic rats; and (2) To evaluate the effect of inactivating the amygdala nuclei on the nociceptive response, anxiety and depressive behaviors, motor activity, and plasma stress hormones in animals with neuropathic pain. Thus, mechanical hyperalgesia and allodynia, and the pattern of c-Fos staining in the amygdala subnuclei were evaluated in rats with chronic constriction of the sciatic nerve, as well as sham-operated and naïve rats. Once the amygdala subnuclei involved in neuropathic pain response were defined, those subnuclei were pharmacological inactivated. The effect of muscimol inactivation on the nociceptive response (hyperalgesia and allodynia), anxiety (elevated plus-maze), depressive-like behavior (forced swim test), motor activity (open field), and plasma stress hormone levels (corticosterone and adrenocorticotropic hormone) were evaluated in sham-operated and neuropathic animals. The results showed that the anterior and posterior portions of the BLA and the central portion of the CeA are involved in controlling neuropathic pain. The inactivation of these nuclei reversed hyperalgesia, allodynia and depressive-like behavior in animals with peripheral neuropathy. Taken together, our findings improve our understanding of the neurocircuitry involved in persistent pain and the roles of specific amygdala subnuclei in the modulation of neuropathic pain, including the neurocircuitry that processes the affective-motivational component of pain.

## Introduction

Neuropathic pain is a complex chronic pain state caused by a lesion or dysfunction of the somatosensory nervous system^1,2^. Animal models can provide information about the course, etiology and treatment for neuropathic pain, and several phenomenon associated with neuropathic pain, such as hyperalgesia, allodynia and spontaneous pain, have been replicated in animal models^1^. One of the models most widely used to mimic peripheral nerve injury is the chronic constriction injury (CCI) model^3^. Neuropathic pain consists of a multidimensional and multifactorial experience that reflects the functional integration and modification of structures of the limbic and cortical systems responsible for the perception of pain and the responses, including their sensory, cognitive and affective aspects, generated as a result of the injury^4^. In addition, patients with chronic pain suffer from changes in the neuroendocrine axis, including abnormal levels of cortisol, which are modulated in important ways by limbic system structures that act to integrate pain control and stress regulation^5–7^. However, the literature regarding cortisol under chronic pain remains contradictory; studies have reported changes in cortisol levels, as well as no changes and alterations in the hypothalamic pituitary adrenal (HPA) axis depending on the type of chronic pain^5–11^.

It has been shown that more than 50% of patients who suffer from chronic pain have comorbid depression and anxiety^12,13^. It has been hypothesized that patients with long-term chronic pain will develop deficits in cognition, anxiety disorders and depression due to alterations in the anatomical integrity and functions of brain regions involved in pain and emotional control^14^.

Accumulating evidence suggests that the amygdala is a key neural substrate for the interactions between pain and negative affective states^15–20^. The amygdala is responsible for modulating emotional responses, such as fear, sadness, depression and anxiety, and has emerged as a structure critical to pain chronicity that contributes to the affective-motivational and cognitive aspects of pain^15,16,21^. The amygdala (or amygdaloid complex) is a structure located in the medial temporal lobe that is formed by a heterogeneous group of nuclei, each of which has a different pattern of cytoarchitecture, neurochemistry, connectivity and functionality^22–25^ have proposed the following classification for the amygdala nuclei: (1) the basolateral nucleus (BLA) is subdivided into anterior (BLAa) and posterior (BLP) portions, (2) the lateral nucleus (LA) has dorsolateral (LAdl) and ventrolateral (LAvl) subdivisions, and (3) the central nucleus (CeA) is formed by medial (CeAm), lateral (CeAl) and central (CeAc) portions. The LA/BLA complex is an important entry point for motor control, sensory, visual, auditory, olfactory and somatosensory systems, including those involved in pain, and receives inputs from the thalamus, insular and sensory cortices, anterior cingulate cortex and medial prefrontal cortex^20,22,23^. The CeA is considered an output area that is responsible for the expression of emotional and physiological responses as well as pain-related functions. It has projections to the hypothalamus and other forebrain structures, including the periaqueductal gray, which plays an important role in controlling the descending analgesic system^20,22,23^.

Several clinical and experimental studies have reported that amygdala nuclei are involved in the regulation of neuropathic pain^26–29^. The activity and neuroplasticity of the amygdala are increased under neuropathic pain conditions^26,28^, and the LA/BLA-CeA circuit is responsible for generating and modulating chronic pain-related behaviors/neuroplasticity^15,18,30^. Taken all together, the currently available data indicate that the amygdala is considered a pain-modulating center in which affective-motivational and sensory-dissociative information is integrated. Here, we use an experimental model of peripheral neuropathy in which we focus on the amygdala to improve our understanding of the neurocircuitry involved in the modulation of neuropathic pain and how it controls the affective-motivational aspects of pain.

To achieve this, we assess the roles of amygdala nuclei in the maintenance and neuromodulation of neuropathic pain and in anxiety and depressive behaviors. Based on theories of pain chronicity and the functions of the amygdala, we hypothesized that the LA, BLA and CeA are involved in controlling neuropathic pain and modulating nociceptive and emotional information. However, a detailed assessment of the amygdala subdivisions that affect neuropathic pain, affective behaviors and endocrinology approach has not been previously reported. Hence, our specific goals were: (1) To evaluate the nociceptive responses and the patterns of c-Fos activation of the CeA, BLA and LA in neuropathic rats; (2) To evaluate the effect of inactivating the amygdala nuclei on the nociceptive response, anxiety and depressive behaviors, motor activity, and plasma stress hormones in animals with neuropathic pain. The pattern of neuronal activation was evaluated using the expression of the immediate early gene c-Fos, which is a widely used tool to map neuronal activation under different physiological and non-physiological conditions^31,32^. c-Fos expression has been used to study the neural circuitry underlying nociception^31,33^.

## Results

### Neuropathic pain, amygdala subnuclei and c-Fos labeling

**Figure 1:**
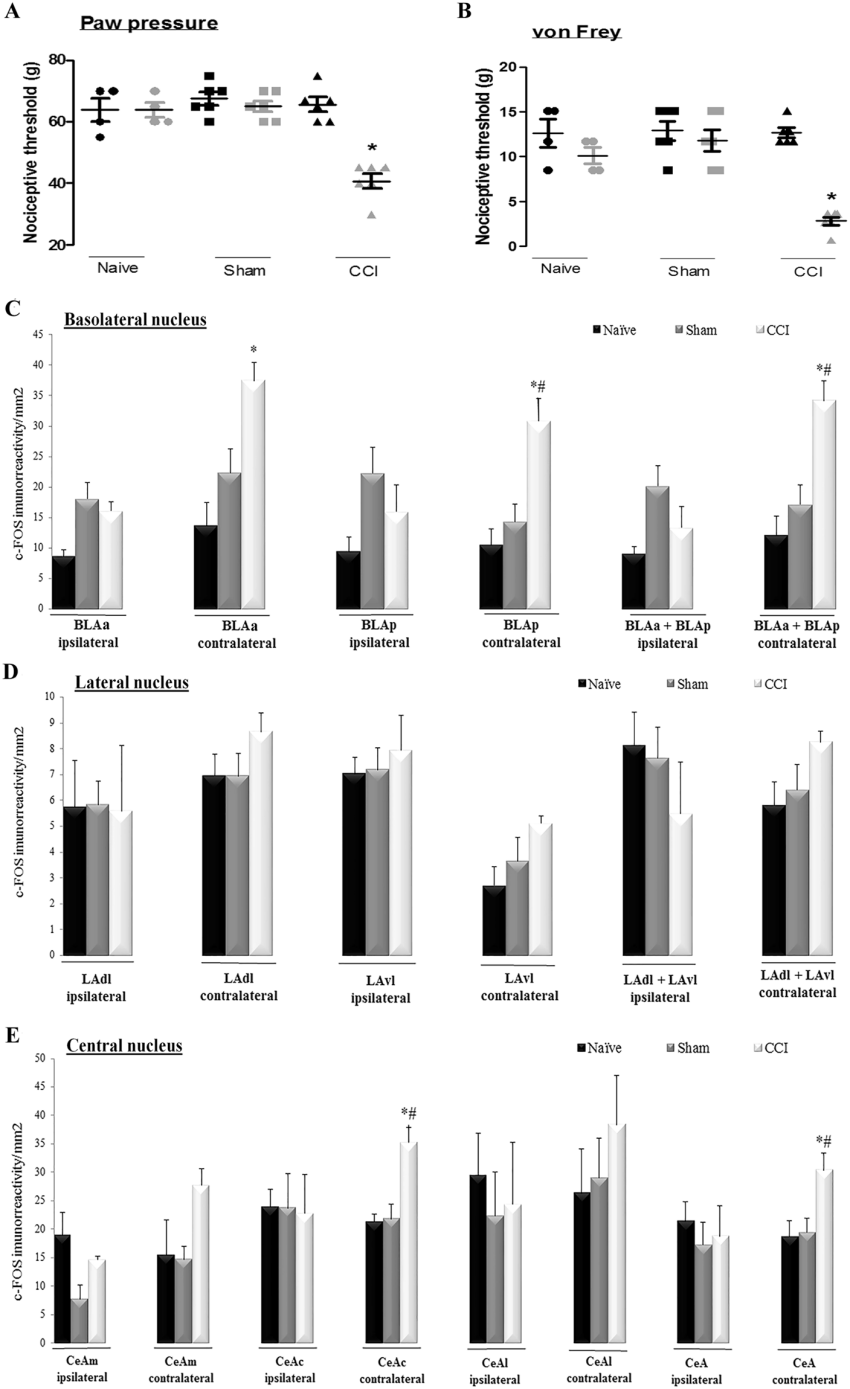
Evaluation of mechanical hyperalgesia in the right paw of naive rats (n = 4), sham-operated rats (Sham, n = 6) and animals submitted to chronic constriction injury of the sciatic nerve (CCI, n = 6). Measurements were taken before (basal measurement, M1 - black) and after the surgical procedure or equivalent (measurement 2, M2 - gray) (**A**). Evaluation of mechanical allodynia using the von Frey filaments in the right paw of naive, sham and CCI rats (**B**). Mean density of c-Fos-IR in naive, sham and CCI rats was evaluated in the basolateral nucleus (BLA) of the ipsilateral (corresponding to the right) or contralateral (corresponding to the left) amygdala. Measurements were obtained for the anterior (BLAa) and posterior (BLP) portions and their average (BLAa + BLP) for the ipsilateral (corresponding to the right) and contralateral (corresponding to the left) amygdala (**C**). The mean density of c-Fos-IR was evaluated in naive, sham and CCI rats in the lateral nucleus (LA) of the ipsilateral (corresponding to the right) or contralateral (corresponding to the left) amygdala. Measurements were obtained for the dorsolateral (LAdl) and ventrolateral (LAvl) portions of the nucleus and their average (LAdl + LAvl) (**D**). The mean density of c-Fos-IR was evaluated in naive, sham and CCI rats in the central nucleus of the amygdala (CeA) in both the ipsilateral (corresponding to the right) and contralateral (corresponding to the left) hemispheres. Measurements were obtained for the medial (CeAm), central (CeAc) and lateral (CeAl) portions and their average (CeAm + CeAc + CeAl) (**E**). The data shown represent the means ± SEM, or dot plot for the behavioral data. *P < 0.05 vs. all other measurements, ^#^P < 0.05 vs. sham group performers. IR: immunoreactivity.

Newman-Keuls post hoc test showed that peripheral neuropathy (CCI) induced a significant decrease in the nociceptive threshold, inducing mechanical hyperalgesia (F_(5,20)_ = 9.55, p = 0.0001; Fig. 1A) and mechanical allodynia (F_(5,20)_ = 7.67, p = 0.0004; Fig. 1B) in the right hind paw in comparison with the baseline measurements. There were no changes in the nociceptive threshold among the measurements taken at or after baseline in the sham and naive rats (Fig. 1A,B).

The BLA, LA, CeA and their subdivisions were evaluated for c-Fos-IR, as shown in the photomicrography in Supplementary Figure (Fig. S1). Immunohistochemistry was performed on the sections obtained from animals that had previously been evaluated in the nociceptive tests. The absolute conditions under which the tests were carried out included the absence of nociception in naive and sham animals and the presence of hyperalgesia and allodynia in animals with peripheral neuropathy. c-Fos-IR was evaluated in the BLA in the BLAa and BLP in both the ipsilateral and contralateral side (or in the corresponding regions in control animals). There were no differences among the groups in results observed in the ipsilateral side, specifically in the BLAa (F_(1,4)_ = 4.31, P = 0.051), BLP (F_(1,4)_ = 2.69, P = 0.12) and the whole BLA (BLAa + BLP − F_(1,4)_ = 3.82, P = 0.08) (Fig. 1C). Newman-Keuls post hoc test showed that the CCI animals showed more labeling for c-Fos-IR in the contralateral BLA (F_(1,4)_ = 5.32, P = 0.003) and BLP (F_(1,4)_ = 9.62, P = 0.006), or the entire BLA (BLAa + BLP − F_(1,4)_ = 7.90, P = 0.008) than was observed in the naive and sham animals (Fig. 1C).

In the LA, both the LAdl and LAvl were evaluated. There were no differences in c-Fos-IR among the groups in the ipsilateral LAdl (F_(1,3)_ = 0.00, P = 0.99) and LAvl (F_(1,10)_ = 0.20, P = 0.81), or the entire LA (F_(1,9)_ = 0.74, P = 0.50) (Fig. 1D). There were also no differences among the groups in the contralateral LAdl (F_(1,10) _= 0.94, P = 0.42) and LAvl (F_(1,10)_ = 1.57, P = 0.25), or the entire LA (LAdl + LAvl − F_(1,10)_ = 1.34, P = 0.30) (Fig. 1D).

In the CeA nucleus, we evaluated the CeAm, CeAl and CeAc. There were no differences among the groups in the ipsilateral side CeAm (F_(1,9)_ = 3.03, P = 0.098), CeAl (F_(1,10)_ = 0.22, P = 0.80), CeAc (F_(1,10)_ = 0.00, P = 0.99)) or the whole CeA (CeAm + CeAl + CeAc: F_(1,10)_ = 0.20, P = 0.81) (Fig. 1E). Newman-Keuls post hoc test showed that c-Fos-IR was higher in the contralateral CeAc (F_(1,10)_ = 6.86, P = 0.006) and whole CeA (F_(1,10)_ = 3.73, P = 0.0003) in the CCI rats. There were no differences in the other subdivisions: CeAm (F_(1,10)_ = 2.66, P = 0.11) and CeAl (F_(1,10)_ = 0.74, P = 0.66) (Fig. 1E).

### Peripheral neuropathy, inactivation of amygdala nuclei and sensory and emotional responses

#### Histological data

**Figure 2:**
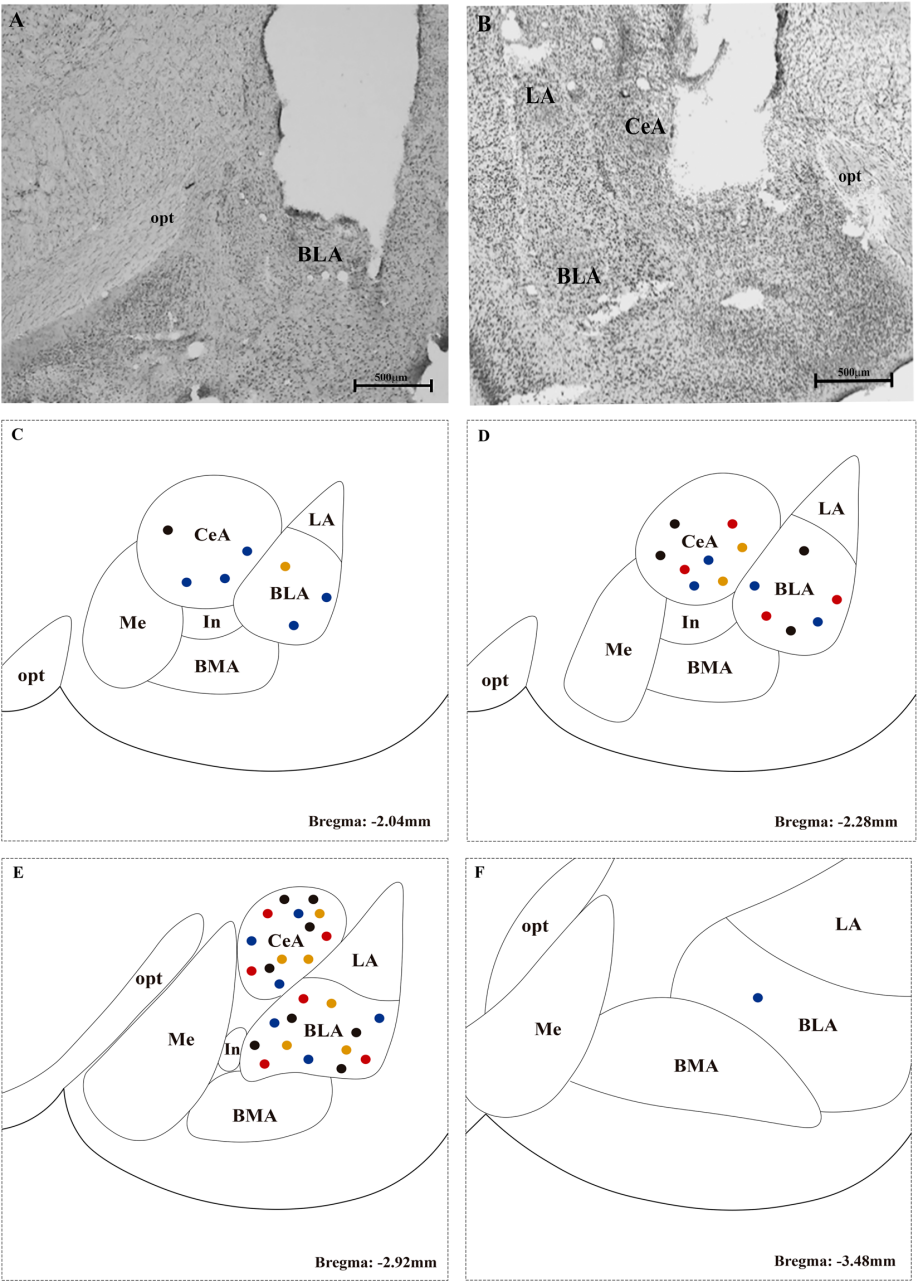
Representative photomicrographs of sites of injection into the basolateral (BLA) (**A**) and central (CeA) (**B**) nuclei of the amygdala. A representation of the microinjections sites along Bregma −2.04 (**C**), −2.28 (**D**), −2.92 (**E**) and −3.48 mm (**F**) is shown for BLA- and CeA-targeted injections. Each dot indicates the site of injection corresponding to a different group. FOP saline: black dots; FOP muscimol: orange dots; CCI saline: red dots, and CCI muscimol: blue dots. Scale bars represent 500 μm in all photographs. BMA: basomedial nucleus of the amygdala; In: intercalated nucleus of the amygdala; Me: medial nucleus of amygdala; opt: optic tract.

After histological evaluation, only animals with a cannula targeted to the BLA and CeA were included in the study. Of the 73 rats that were submitted to stereotactic surgery, 49 were included in the study (23 had a bilateral cannula targeted to the BLA, and 26 were targeted to the CeA). The remaining rats were excluded because they died after surgery (n = 3), were targeted only unilaterally (n = 14) or had a cannula outside the target (n = 7). Figure 2 shows a photomicrography of the injections targeting the BLA (Fig. 2A) and CeA (Fig. 2B). The locations of the microinjections along Bregma from −2.04 to −3.48 mm are shown (Fig. 2C–F).

#### Nociceptive response

**Figure 3:**
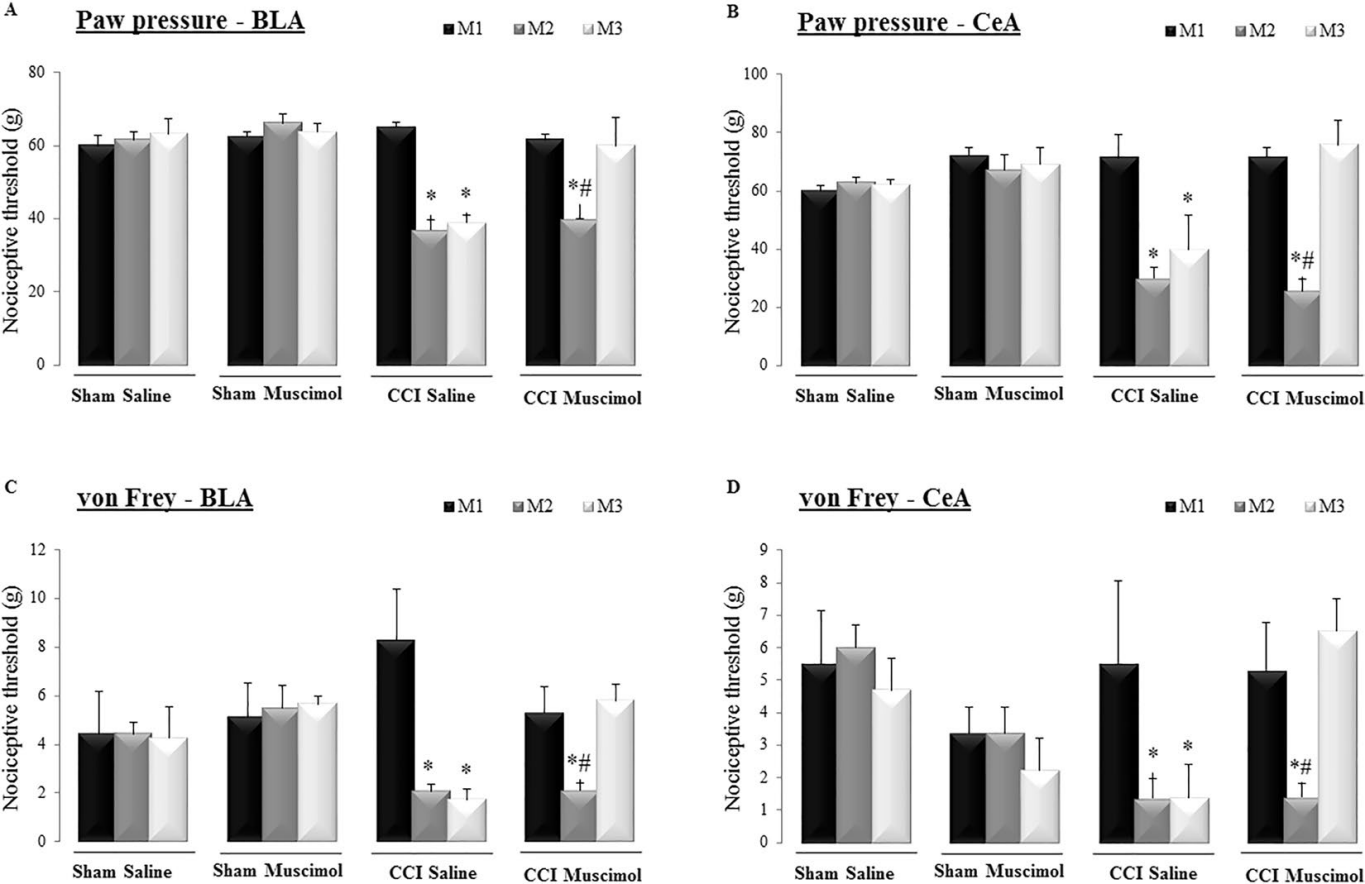
Evaluation of mechanical hyperalgesia in the right hind paw of naive and sham-operated rats (Sham) and animals submitted to chronic constriction injury (CCI) of the sciatic nerve. Basal measurements (M1 - black), measurement 2 (M2 - dark gray) and those taken after the microinjection (M3 - light gray) of saline or muscimol targeted to the basolateral (BLA) (**A**) or central (CeA) (**B**) nucleus of the amygdala are shown. Mechanical allodynia was evaluated using von Frey filaments in the right hind paw at M1, M2 and M3 in naive, sham and CCI animals with cannulas targeted to the bilateral BLA (**C**) or CeA (**D**) nuclei of the amygdala. *P < 0.05 vs. measurement 1 (M1); ^#^P < 0.05 vs. CCI rats administered muscimol (M3), sham rats microinjected with saline (n = 6/BLA and n = 7/CeA) or muscimol (n = 4/BLA and n = 5/CeA), or CCI rats microinjected with saline (n = 5 for BLA and CeA) or muscimol (n = 8 for BLA and CeA).

The animals were evaluated using nociceptive tests before (M1), at 14 days after the surgical sham or CCI procedure (M2) and at 30 min after the administration of saline or muscimol into the BLA or CeA (M3). In the CCI rats, the nociceptive threshold was lower after surgery than at M1 (Fig. 3A). Newman-Keuls post hoc test showed that the local administration of muscimol into the BLA (Factor 1: surgery, F_(2,12)_ = 9.48, P = 0.000002; Factor 2: drugs F_(2,12)_ = 89.58, P = 0.000000; Interaction: F_(2,12)_ = 6.82, P = 0.000000; Fig. 3A) or the CeA (Factor 1: surgery, F_(2,12)_ = 7.91, P = 0.00001; Factor 2: drugs, F_(2,12)_ = 63.91, P = 0.0000; Interaction: F_(2,12)_ = 3.85, P = 0.00005; Fig. 3B) reversed the mechanical hyperalgesia observed in the CCI animals. In the sham rats, no alterations in the threshold measurements were observed between the baseline measurements and those taken after the administration of saline or muscimol (Fig. 3A,B). There was no difference in the nociceptive threshold of the contralateral, intact paw (left paw) (see Supplementary Figure - Fig. S2) in rats with a BLA cannula (Factor 1: F_(2,12)_ = 2.71, P = 0.033; Factor 2: F_(2,12)_ = 81.63, P = 0.00; Interaction: F_(2,12)_ = 1.69, P = 0.07) or a CeA cannula (Factor 1: F_(2,12)_ = 0.29, P = 0.87; Factor 2: F_(2,12)_ = 73.58, P = 0.0000; Interaction: F_(2,12)_ = 0.15, P = 0.99)).

In the von Frey test, in the sham group, there was no difference in the nociceptive response between the initial measurement and that taken after the muscimol injection in the BLA (Fig. 3C) or in the CeA (Fig. 3D). In the CCI rats, Newman-Keuls post hoc test showed that the nociceptive threshold was lower after surgery than at either M1 or after the administration of muscimol in the BLA (Factor 1: F_(2,12)_ = 2,04, P = 0,09; Factor 2: F_(2,12)_ = 48,47, P = 0,000000; Interaction: F_(2,12)_ = 2,60, P = 0,01; Fig. 3C) or CeA (Factor 1: F_(2,12)_ = 5,30, P = 0,04; Factor 2: F(2,12) = 37,00, P = 0,00; Interaction: F_(2,12)_ = 6,82, P = 0,03; Fig. 3D). Hence, muscimol reversed mechanical allodynia in CCI rats.

#### Motor activity

When the BLA and CeA were targeted, there were no differences among the sham and CCI groups treated with saline or muscimol, regardless of the total distance in the periphery (for cannulas targeted to the BLA, Factor 1: F_(1,19)_ = 2.29, P = 0.14; Factor 2: F_(1,19)_ = 0.31, P = 0.58; Interaction: F_(1,19)_ = 1.83, P = 0.19; for cannulas targeted to the CeA, Factor 1: F_(1,19)_ = 2.29, P = 0.14; Factor 2: F_(1,19)_ = 0,31, P = 0,58; Interaction: F_(1,19)_ = 1.83, P = 0.19); the total distance to the central area (when the BLA was targeted, Factor 1: F_(1,19)_ = 1.51, P = 0.23; Factor 2: F_(1,19)_ = 0.06, P = 0.79; Interaction: F_(1,19)_ = 0.00, P = 0.95; and when the CeA was targeted, Factor 1: F_(1,19)_ = 1.51, P = 0.23; Factor 2: F_(1,19)_ = 0.06, P = 0.79; Interaction: F_(1,19)_ = 0.00, P = 0.95) and the total distance (BLA target, Factor 1: F_(1,19)_ = 2.46, P = 0.13; Factor 2: F_(1,19)_ = 0.18, P = 0.67; Interaction: F_(1,19)_ = 1.35, P = 0.25; and CeA target, Factor 1: F_(1,19)_ = 2.46, P = 0.13; Factor 2: F_(1,19)_ = 0.18, P = 0.67; Interaction: F_(1,19)_ = 1.35, P = 0.25). Data for additional measurements recorded in the open field tests are shown in Supplementary Table 1.

#### Anxiety-like behavior

In animals with a cannula targeted to the BLA or CeA, there were no differences in classic anxiety measurements among the sham and CCI groups treated with saline or muscimol. These measurements included entries into the open arms (BLA target, Factor 1: F_(1,19)_ = 2.02, P = 0.17; Factor 2: F_(1,19)_ = 7.87, P = 0.27; Interaction: F_(1,19)_ = 2.73, P = 0.11; and CeA target, Factor 1: F_(1,23)_ = 2.94, P = 0.09; Factor 2: F_(1,23)_ = 4.67, P = 0.04; Interaction: F_(1,23)_ = 0.09, P = 0.76), entries in to the open arm extremities (BLA target, Factor 1: F_(1,19)_ = 0.14, P = 0.70; Factor 2: F_(1,19)_ = 0.02, P = 0.87; Interaction: F_(1,19)_ = 2.65, P = 0.11; and CeA target, Factor 1: F_(1,23)_ = 0.47, P = 0.49; Factor 2: F_(1,23)_ = 1.36, P = 0.25; Interaction: F_(1,23)_ = 3.92, P = 0.05) and time spent in the open arms (BLA target, Factor 1: F_(1,19)_ = 0.76, P = 0.39; Factor 2: F_(1,19)_ = 0.01, P = 0.91; Interaction: F_(1,19)_ = 1.54, P = 0.22; and CeA target, Factor 1: F_(1,22)_ = 0.02, P = 0.87; Factor 2: F_(1,22)_ = 4.51, P = 0.04; Interaction: F_(1,22)_ = 0.13, P = 0.72) and in the open arm extremities (BLA target, Factor 1: F_(1,19)_ = 0.20, P = 0.65; Factor 2: F_(1,19)_ = 0.28, P = 0.60; Interaction: F_(1,19)_ = 1.49, P = 0.23; and CeA target, Factor 1: F_(1,23)_ = 0.57, P = 0.45; Factor 2: F_(1,23)_ = 1.97, P = 0.17; Interaction: F_(1,23)_ = 0.04, P = 0.84). Data from additional measurements of observed behaviors in the elevated plus-maze are shown in Supplementary Table 2.

#### Depressive-like behavior

**Figure 4:**
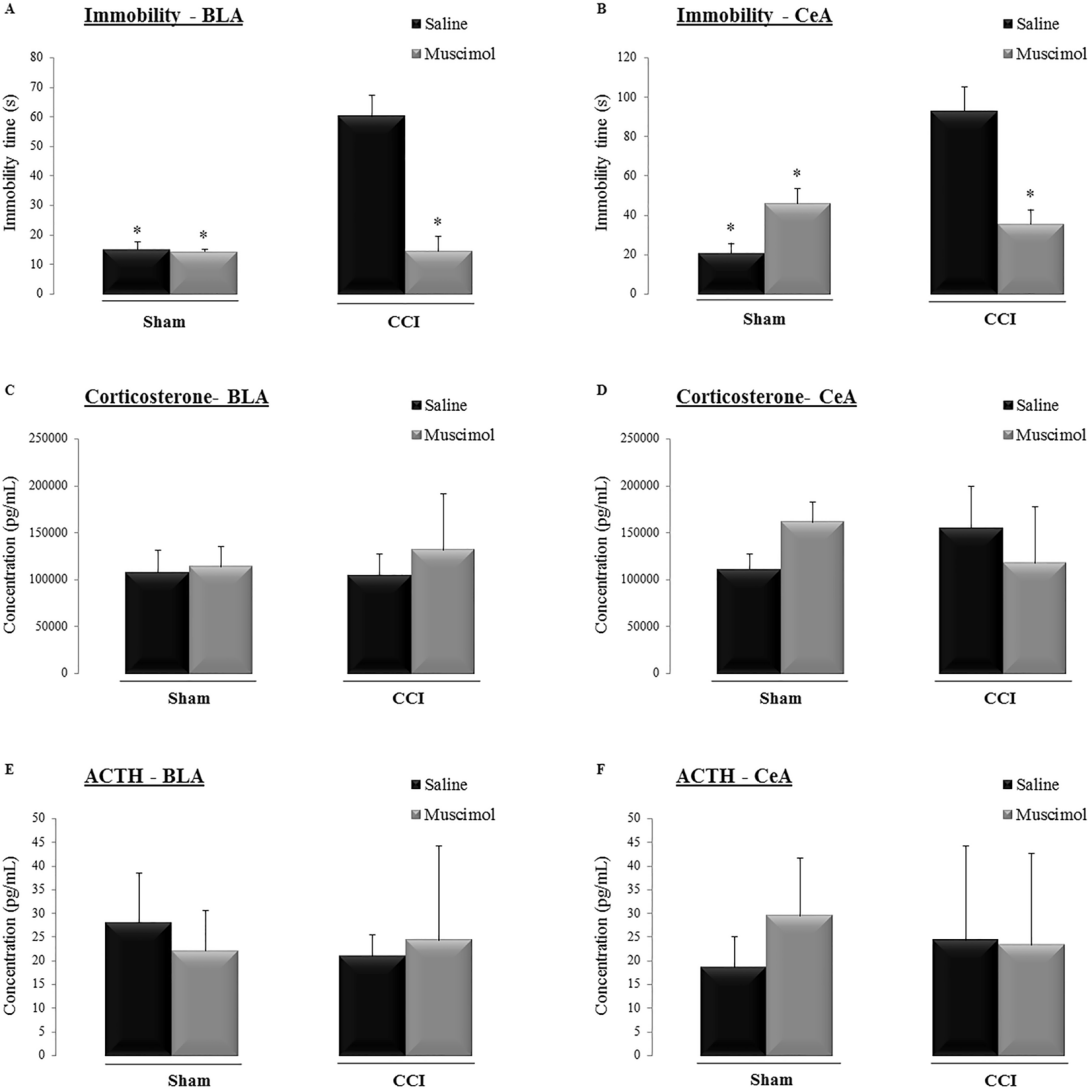
Time spent (s) immobile during the forced swimming test in the sham-operated rats (Sham) and animals submitted to chronic constriction injury (CCI) of the sciatic nerve after the microinjection of saline or muscimol targeted to the basolateral (BLA) (**A**) or central (CeA) (**B**) nucleus of the amygdala. Serum corticosterone levels (pg/mL) were measured in the sham and CCI rats after the microinjection of saline or muscimol into the BLA (**C**) or CeA (**D**) nuclei of the amygdala. Serum adrenocorticotrophic hormone (ACTH) levels (pg/mL) in the sham and CCI rats after the microinjection of saline or muscimol targeted to the BLA (**E**) or CeA (**F**) nuclei of the amygdala. The data shown represent the means ± SEM. *P < 0.05 vs. all other groups. Groups: Sham saline (n = 6/BLA and n = 7/CeA), Sham muscimol (n = 4/BLA and n = 5/CeA), CCI saline (n = 5 for BLA and CeA) and CCI muscimol (n = 8 for BLA and CeA).

Newman-Keuls post hoc test showed that sham animals injected with saline or muscimol had a shorter immobility time than that observed in the CCI animals injected with saline (CCI saline) into either the BLA (Fig. 4A) or CeA (Fig. 4B). Additionally, Newman-Keuls post hoc test indicated that in animals with neuropathic pain, the administration of muscimol (CCI muscimol) resulted in less immobility time than was observed in the CCI saline animals, regardless of which amygdala nuclei was targeted (BLA target, Factor 1: F_(1,20)_ = 20.83, P = 0.0002; Factor 2: F_(1,20)_ = 21.94, P = 0.0001; Interaction: F_(1,20)_ = 20.70, P = 0.0002; Fig. 4A; and CeA target, Factor 1: F_(1,21)_ = 11.67, P = 0.003; Factor 2: F_(1,21)_ = 3.20, P = 0.09; Interaction: F_(1,21)_ = 20.89, P = 0.0002; Fig. 4B).

#### Corticosterone and ACTH levels

The plasma levels of corticosterone and ACTH were quantified in sham and CCI rats treated with saline or muscimol for both targets (the BLA or CeA). There were no differences among the groups in corticosterone levels (BLA target, Factor 1: F_(1,20)_ = 0.20, P = 0.64; Factor 2: F_(1,20)_ = 1.07, P = 0.31; Interaction: F_(1,20)_ = 0.42, P = 0.52; Fig. 4C; and CeA target, Factor 1: F_(1,21)_ = 0.00, P = 0.99; Factor 2: F_(1,21)_ = 0.14, P = 0.71; Interaction: F_(1,21)_ = 6.14, P = 0.02; Fig. 4D) and ACTH levels (BLA target, Factor 1: F_(1,16)_ = 0.20, P = 0.66; Factor 2: F_(1,16)_ = 0.05, P = 0.81; Interaction: F_(1,16)_ = 0.76, P = 0.39; Fig. 4E; and CeA target, Factor 1: F_(1,17)_ = 0.00, P = 0.97; Factor 2: F_(1,17)_ = 0.54, P = 0.47; Interaction: F_(1,17)_ = 0.78, P = 0.38; Fig. 4F).

## Discussion

First, peripheral neuropathy induced mechanical hyperalgesia and allodynia at 14 days after the surgical procedure (CCI) but not in the control, naive or sham rats. The amygdala nuclei that are most importantly involved in neuropathic pain are the BLA (BLAa and BLP) and CeA (CeAc). The inactivation of both of these nuclei reversed hyperalgesia, allodynia and depressive-like behavior in animals with neuropathic pain.

The amygdala is a critical link between neuropathic pain and the affective-motivational component of pain^15,19,25^. An important contribution of our work is our detailed assessment of the roles of the subdivisions of the BLA (subdivided into the BLAa and BLP), LA (subdivided into the LAdl and LAvl) and CeA (subdivided into the CeAm, CeAl and CeAc) in neuropathic pain. The amygdala is a highly complex structure with subdivisions that have previously been shown to play different and opposing functions^19,23,34,35^. As far as we know, no previous studies have focused on the unique roles of these subdivisions of amygdala nuclei in neuropathic pain. Patients with chronic pain and anxiety have abnormal amygdala connectivity. Specifically, BLA-predominant amygdala connections are blocked in patients with chronic pain, supporting that the BLA links pain and emotion^36^. Another amygdala nucleus, the LA, receives the main sensory, visual, auditory, somatosensory and olfactory inputs^20,37^. Martin *et al*.^38^ reported that the LA mediates the anti-allodynic effects of heroin after peripheral nerve injury. One possible reason for the difference from our work could be the anatomical nomenclature; the LA, basal and accessory-basal nuclei are sometimes viewed as a single structure called the BLA complex^23^. In support of our hypothesis, previous studies have shown that the BLA complex plays a role in pain^15,39^. Regarding the CeA, it has a medial, lateral and central portion, each of which is defined based on its unique cytoarchitecture, neurochemistry and connectivity^19,35^. Previous studies have shown that the CeAc plays a key role in pain^15,40,41^. Furthermore, in support of our findings, a previous study utilizing whole-cell patch-clamp recordings in the CeAc showed that the activation of this area contributes to pain-related synaptic facilitation^42^. Previous studies have also found that a reduction of GABAergic inhibition via intra-CeA administration of muscimol^43^ or diazepam^16^ reversed neuropathic pain in CCI rats. The same anti-nociceptive effect has been reported after injection of a mu opioid agonist into the BLA^44,45^. In summary, our study suggests that distinct portions of amygdala nuclei are activated by neuropathic pain, and that the differences among the subnuclei might contribute to the complexity of persistent pain behaviors^2,41^. In terms of allodynia, one possible explanation for the baseline differences between the experiments could be that the rats in the inactivation experiment received cannula implantation targeting the amygdala, which is highly involved in the pain control mechanism^19,25^.

Neuropathic pain induces mood disorders, such as depression and anxiety, in both patients^12,13^ and animal models^4,46–48^. Neuroimaging studies have suggested that hyperactivation of the amygdala increases the developing of depression^49^, possibly due to neuroplasticity of the amygdala^28,50^. Therefore, the BLA and CeA subnuclei might modulate emotional-affective pain behaviors^39^. In support of this hypothesis, a postmortem study suggested that depressed subjects may have increased BLA activity^51^. Additionally, neuropathic rats that exhibited depression-like behavior also showed increased cell proliferation in the CeA and BLA^28^. Electroconvulsive therapy produces neuroplasticity in the BLA in major depressive patients^52^. Taken together with the results in the literature, our data indicate that the CeA and BLA are the main modulators of depression-associated persistent pain. Some of the possible mechanisms responsible for the depressive-like behavior could be activation of corticotrophin-releasing factor (CRF)/CRF 1 signaling in the BLA, sensitization of BLA neurons concomitantly with an augmentation of long-term potentiation at BLA-CeA synapses, and/or abnormalities in glutamatergic neurotransmission^53–55^.

To assess locomotor and exploratory activity, animals were tested in the open field test^56,57^. In accordance with our results, neither the CCI model^28^ nor amygdala inactivation affected motor activity^58,59^. However, other studies have shown that CCI decreases locomotor activity^60,61^. One potential reason for this difference may be differences in the experimental design, in that CCI animals show fewer exploratory behaviors when evaluated at 5 weeks after surgery^60^ or after they were re-tested in the open field^61^.

Regarding anxiety, previous studies have shown that anxiety-like behavior is a common feature in animal models of neuropathic pain^47,62^. The reason for this discrepancy with our results could be the use of different animal models or the time points at which evaluations were performed because in CCI models, anxiety features are observed only at 4 weeks after the induction of neuropathy^63^. Furthermore, some authors have reported that microinjecting muscimol into the amygdala has either an anxiolytic-like effect^64^ or no effect^65^. In the presence of neuropathic conditions, it has been proposed that GABAergic modulation occurring in the CeA nucleus could regulate neuropathic pain-related anxiety-like behaviors^16^. In this sense, our interpretation of these data is that the time chosen to assess anxiety-like behaviors may not have been appropriate for detecting this symptom in animals with neuropathic pain.

In terms of neuroendocrine factors, patients with chronic pain often exhibit HPA disorders, including abnormal levels of cortisol^5,6^. However, in support of our data, in the CCI model, basal HPA axis function is unchanged^8,9^ and appears to be independent of the affective consequences of neuropathic pain^4^.

In terms of the technical limitations that result from the invasiveness of the microinjection procedure, specifically due to the size of the cannula and the pattern of spread of muscimol in the amygdala, it is not possible to only target specific subnuclei of the amygdala, including the BLAa, BLP and CeAc. Furthermore, although the behavioral tests were counterbalanced across all rats to control for potential bias, we could not discard that one of the behavioral tests might have impacted the other or might have produced unwanted variability in the performance of the animals, consequently affecting the blood analyses. Drug spread was not evaluated in the study; however, an autoradiographic study^66^ estimated the average muscimol spread to be 1.7 mm.

## Conclusion

The results of our study provide a better understanding of the mechanisms involved in the maintenance and complexity of neuropathic pain. It is important to emphasize that our main contribution in the neuropathic field is that we have connected the amygdala subnuclei, the CeA and BLA, with the modulation of depressive-like behaviors.

## Methods

### Animals

Eighty-six male Wistar rats (180–200 g) obtained from the animal facility of the University of São Paulo were used as subjects. The animals were housed three rats per cage in polypropylene cages (40 × 34 × 17 cm) containing wood shavings for at least five days before the experimental procedures. The cages were kept in a room with a stable, controlled ambient temperature (22 ± 2 °C) and a 12:12 dark/light cycle (lights on at 07:00 h). The animals had free access to water and rat chow pellets throughout the experiment. The experiments were performed in compliance with the guidelines for the ethical use of animals in research involving pain and nociception^67^ and the recommendations of the Brazilian Society of Neuroscience and Behavior, which, in turn, are based on the US National Institutes of Health Guide for the Care and Use of Laboratory Animals. Furthermore, they were reported in accordance with the ARRIVE guidelines (http://www.nc3rs.org.uk/arrive-guidelines). The study was approved by the Ethics Committee on the Use of Animals at Hospital Sírio Libanês (protocol number CEUA 2014-07).

### Induction of peripheral neuropathy

The rats were anesthetized with isoflurane (approximately 4% for induction and 2% for maintenance, oxygen 100%), injected with antibiotics (0.1 mL/kg, Zoetis, NJ, USA) and subjected to sciatic nerve CCI as previously described^68^. Using a sharp and blunt dissection instrument, the right sciatic nerve was exposed at the mid-thigh level. Proximal to the sciatic trifurcation (approximately 7 mm), four ligatures were loosely tied (1–1.5 mm apart) around the nerve using 4.0 catgut chrome wire. The skin was closed with 4.0 nylon wire. Sham rats were subjected to the same surgical procedure but without constriction of the nerve, and naive rats were not submitted to any surgical procedure.

### Implantation of an intracerebral cannula

Under the same inhalational general anesthesia protocol, animals were fixed in a stereotaxic frame (David Kopf, CA, USA) and then received local anesthesia (Xylestesin 2% + Hemitartarate of Norepinephrine 1:50.000; 0.14 mL/100 g, Cristalia, SP, BRA). The upper incisor bar was set at 2.5 mm below the interaural line so that the skull was horizontal between Bregma and lambda. Using a rat brain atlas^69^ for guidance, guide cannulas were bilaterally placed in each animal at either the BLA (AP = 2.5 mm; ML = 5.1 mm; VD7.7 mm) or CeA (AP = 2.4 mm, ML = 4.0 mm, DV = 7.0 mm). At the end of the surgical procedure, the animals were observed until they fully recovered from the anesthesia.

### Drug administration

Saline or muscimol (0.5 μg/0.2 μL) were microinjected into the amygdala nuclei (the BLA or CeA). Muscimol provides an efficient, reversible inactivation effect and was chosen based on the results of previous studies^70–73^. The injection was performed using a thin dental needle (0.3 mm outer diameter) connected to a 5 μL Hamilton syringe by a polyethylene tube. The injection needle was introduced into and placed 1 mm below the guide cannula. The solutions were injected into the amygdala nuclei (0.2 μL/min) using an infusion pump (Harvard Apparatus, MA, USA). The displacement of an air bubble inside a polyethylene catheter (PE-10; Becton–Dickinson, NJ, USA) connecting the syringe needle to the intracerebral needle was used to monitor the microinjection. After the injection, the needle was held for an additional 1 min to maximize diffusion.

### Assessment of the nociceptive response

Nociceptive tests were performed before any intervention (baseline measurement – M1) and on the 14th day after the surgical procedure or equivalent (M2 - CCI, sham and naive groups). Additionally, the measurements were re-evaluated after the microinjection of saline or muscimol (M3). The results were analyzed by comparing the initial and final measurements to each other. The investigators were blind to group identification.

#### Evaluation of mechanical hyperalgesia

The rat paw pressure test^74^, which employs the use of a pressure apparatus (Insight Ltda., SP, BRA), was used to analyze mechanical hyperalgesia. Briefly, the test consisted of the application of a force of increasing magnitude (up to 16 g/s) to the right hind paw. The force (in grams) required to induce a withdrawal response was defined as the nociceptive threshold. To reduce stress, the rats were habituated to the apparatus on the day preceding the experiment.

#### Evaluation of mechanical allodynia

Mechanical allodynia was evaluated in accordance with Milligan *et al*.^75^. The rats were placed individually in plastic cages with a wire bottom that allowed the investigator access to their paws. To reduce stress, the rats were habituated to the experimental environment on each of the 2 days preceding testing. On the day of the test, the animals were placed in the cages 15 min before the beginning of each measurement period. Briefly, mechanical allodynia was evaluated as the paw withdrawal threshold in response to mechanical stimuli defined by a series of von Frey filaments (Semmes–Weinstein monofilaments, Stoelting, IL, USA) ranging from 1.08 to 21.09 g. The von Frey filaments were applied through the mesh floor to the mid-plantar surface of the right hind paw. The filaments were applied in ascending order, and the smallest filament that elicited a paw withdrawal response in two consecutive applications was considered to represent the withdrawal threshold.

### Assessment of the affective-motivational response

Affective-motivational behaviors were evaluated in the CCI and sham animals in comparison to the animals administered saline or muscimol into the amygdaloid nuclei. The animals were microinjected and then tested in an elevated plus-maze and forced swim tests.

#### Evaluation of anxiety-like behaviors

The animals were tested in an elevated plus-maze, a classic method used to assess anxiety-like behavior^76^. The maze details have previously been described in detail^77^. Briefly, it consists of two open arms (50 × 10 cm) crossed at right angles with two opposing closed arms of the same size enclosed by walls 40 cm high. The maze was elevated 50 cm above the floor, and a rim of Plexiglas (0.5 cm high) surrounded the perimeter of the open arms. For each 5-min test, each rat was gently placed in the central area with its nose facing one of the closed arms and allowed to explore the maze. Before the next rat was tested, the maze was cleaned with a 5% ethanol solution and dried with a cloth. All sessions were recorded using a video camera, and the following behaviors were blindly analyzed: (a) head dipping: dipping the head below the level of the maze floor; (b) stretching: the animal stretched to its full length using its forepaws (the hind paws were maintained in the same place) and then turned back to its previous position; (c) rearing: partial or total raising on the hind limbs; (d) sniffing: horizontal head movements in any direction, including sniffing the maze floor and walls; and (e) grooming: species-specific behavioral sequences, including cleaning of any part of the body surface or fur with the tongue, teeth, and/or forepaws.

#### Evaluation of depressive-like behavior

The forced swimming test is one of the most commonly used methods for assessing depressive-like behavior. In this test, the time spent immobile is measured^78^. The rats were individually placed in a cylindrical tank (30 cm diameter × 60 cm height) filled with water at 24 ± 1 °C to 30 cm in depth. The protocol was divided into a 15-min pre-test performed 24 h before a 5-min test. Immobility was scored based on recordings made with a video camera, and all behaviors were blindly scored. The immobility time (in seconds - s) was determined by measuring the time during which no additional activity was observed other than the movements necessary to keep the animal’s head above the surface of the water.

### Assessment of motor activity

Motor behavior was evaluated in open field tests^56^. The open field consists of a 60 cm square of dark gray Formica floor surrounded by 50 cm high walls. During the test, each animal was placed in the center of the apparatus and allowed to freely explore for 5 min, as described previously^79,80^. After the end of the test, the open field was cleaned with 5% ethanol and subsequently dried with a dry cloth. Displacements and the frequency and time spent stretching, rearing, grooming and sniffing were recorded by a video camera, and motor behaviors were blindly scored.

### Perfusion

Because the expression of inducible transcription factor proteins peaks at approximately 1 hour after stimulus induction and fades by 3–4 hours^31^, rats were perfused 1 h after the last behavioral test. For the perfusion, the animals were deeply anesthetized with thiopental (40 mg/kg, i.p., Cristalia) and then transcardially perfused with 0.9% saline solution followed by 4% paraformaldehyde (PFA) dissolved in 0.1 M phosphate buffer (PB, pH 7.4). The brains were removed and post-fixed in PFA for 4 h. They were then incubated in 30% sucrose in PB solution for 48 h at 4 °C and cut into 30 µm-thick sections on a freezing microtome in the frontal plane. One series of sections was mounted on gelatin-coated slides, Nissl-stained and examined using a 10x objective by an observer blinded to the behavioral results. These sections were used as a reference series for cytoarchitectonic purposes or to evaluate cannula placement.

### Immunohistochemistry

The sliced sections were washed in PB and incubated for 12–16 h at 4 °C with rabbit anti-c-Fos primary antibodies (1:20000; Ab-5, Calbiochem, CA, USA) diluted in 0.3% Triton X-100 containing 5% normal donkey serum (Jackson ImmunoResearch, ME, USA). The slides were washed three times for 10 min each with PB and then incubated for 2 h at room temperature with biotinylated secondary antibodies (1:200; donkey anti-rabbit IgG, Jackson ImmunoResearch). They were then incubated with avidin–biotin complexes (1:100; ABC Elite kit, Vector Labs, Burlingame, CA, USA) for 2 h at room temperature. The sections were visualized using a mixture of 0.05% diaminobenzidine tetrahydrochloride (DAB, Sigma-Aldrich, MO, USA) and 0.01% hydrogen peroxide in PB. The DAB reaction was stopped by extensively washing the slides in PB. Sections were mounted on gelatin-coated slides with a glycerol-based mounting medium, air-dried, dehydrated through graded ethanol solutions followed by xylene, and then coverslipped with DPX (Sigma-Aldrich). Finally, images were captured utilizing a light microscope (E1000, Nikon, Melville, NY, USA), and the c-Fos-immunoreactive neurons in amygdala nuclei (BLA, LA and CeA) were evaluated by a blinded observer in 10× images. The subnuclei delineations were defined according to histological landmarks based on the adjoining Nissl-stained sections in the rat brain atlas^69^. Assignment of histological landmarks is a key step for the identification of the subnuclei delineations. The use of the adjoining Nissl-stained sections is a useful tool to identify different nuclei based on the spatial distribution of neurons and defined subpopulations of neurons with greater accurary^81–83^. The observer’s analysis was performed using ImageJ software (National Institutes of Health, MD, USA; http://rsbweb.nih.gov/ij/). The amygdala nuclei were evaluated every 0.24 mm from Bregma −1.80 mm to −4.00 mm.

### Blood samples and stress hormone quantification

During the perfusion, trunk blood samples were obtained from the heart, collected into EDTA-treated vacutainer tubes, immediately placed on regular ice and then centrifuged (15 min at 2500 rpm) to collect plasma. The plasma samples were aliquoted and stored at −80 °C until analyzed. The levels of corticosterone and adrenocorticotropic hormone (ACTH) were measured in duplicate using a multiplex bead-based immunoassay (Rat Stress Hormone Magnetic Bead Panel, RSHMAG-69K) and a Luminex MAGPIX (Millipore Corporation, MA, USA).

### Experimental design

**Figure 5:**
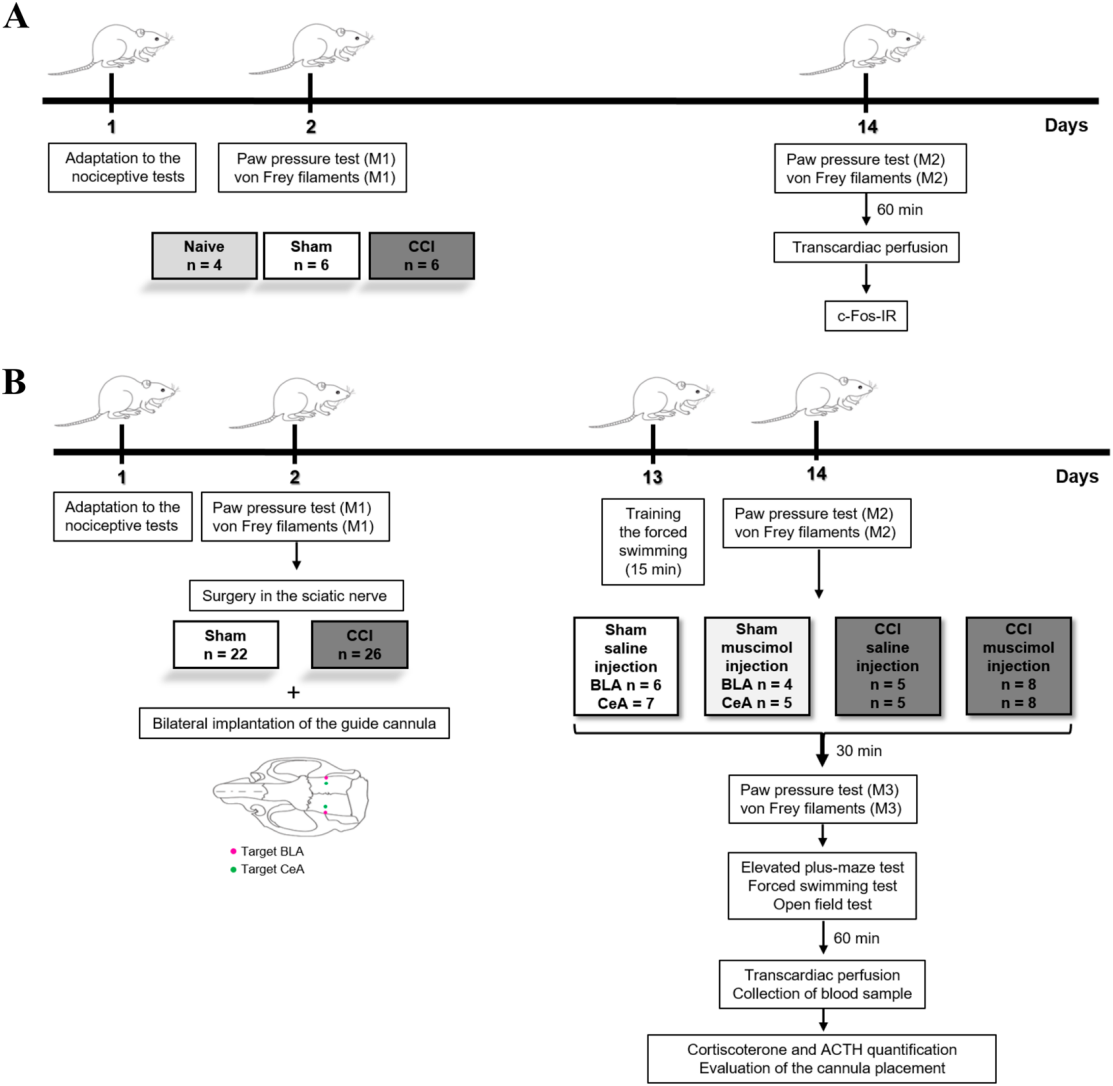
Experimental design of the study. Neuropathic pain, amygdala nuclei and c-Fos labeling (**A**). Peripheral neuropathy, inactivation of amygdala nuclei and sensory and emotional responses (**B**). ACTH: adrenocorticotropic hormone; BLA: basolateral nucleus of the amygdala; CCI: chronic constriction injury of the sciatic nerve; CeA: central nucleus of the amygdala; IR: immunoreactivity; M1: basal measurement; M2: measurement 2; M3: measurement 3; Naive: rats without any surgical procedure; Sham: rats that underwent a false operation.

The animals were adapted to the nociceptive tests. On the next day, baseline measurements (M1) were obtained for the nociceptive tests, and the animals were randomly divided into the following three groups: CCI, sham and naive. At 14 days after the surgery (CCI or sham), all groups were reevaluated in nociceptive tests (M2) to characterize neuropathic pain. At sixty minutes after the last test, the animals were perfused and their tissues collected and processed for immunohistochemical assays to detect c-Fos protein (neuronal activation marker) in the LA, BLA and CeA. Differences among the groups of animals were then compared (Fig. 5A). Once we determined which amygdala nuclei exhibited changes in the pattern of neuronal activation under neuropathic pain conditions, we inactivated the identified nuclei and evaluated the nociceptive response, anxiety and depressive-like behaviors, motor activity, and plasma stress hormones in sham and neuropathic pain animals. In these experiments, the rats were adapted to the nociceptive tests, and baseline measurements (M1) were then taken. The animals were then randomly separated into CCI and sham groups and submitted to the bilateral implantation of the guide cannula in the amygdala nucleus of interest (BLA or CeA). At thirteen days after cannula implantation, the animals were trained in forced swimming for 15 min. On the following day, the nociceptive threshold was reevaluated to characterize neuropathic pain (M2), and animals were then injected with muscimol or saline. At 30 min after injection, the nociceptive threshold (M3) measurements were obtained, and the behavioral tests were performed (elevated plus-maze, forced swimming and open field tests). The behavioral tests were counterbalanced across all rats. At 60 min after the last behavioral test, we perfused the animal and collected a blood sample to quantify corticosterone and ACTH levels. The brain tissues were subsequently cut on a microtome and processed to evaluate cannula placement (Fig. 5B).

### Statistical analyses

The data are reported as the means ± standard errors of the means (SEMs). Considering that our data assumed a normal distribution, c-Fos expression was analyzed using on-way analysis of variance (ANOVA) and one-way repeated measures ANOVA for behavioral data. Data from the elevated plus-maze, open field, forced swimming and stress hormone tests were analyzed using two-way ANOVA (factor 1: type of surgery, sham or CCI; and factor 2: drugs, saline or muscimol). Two-way repeated measures ANOVA was used to evaluate the nociceptive response. All tests were followed by a Newman-Keuls post hoc test. Significance was set at P < 0.05.

## Electronic supplementary material

Supplementary information

## Data Availability

The datasets generated and/or analyzed during the current study are available from the corresponding author upon reasonable request.

## References

[1] Backonja MM (2003). Defining neuropathic pain. Anesth. Analg..

[2] Colloca L (2017). Neuropathic pain. Nat. Rev. Dis. Primers..

[3] Wang LX, Wang ZJ (2003). Animal and cellular models of chronic pain. Adv. Drug. Deliv. Rev..

[4] Yalcin I, Barthas F, Barrot M (2014). Emotional consequences of neuropathic pain: insight from preclinical studies. Neurosci. Biobehav. Rev..

[5] Griep EN (1998). Function of the hypothalamic-pituitary-adrenal axis in patients with fibromyalgia and low back pain. J. Rheumatol..

[6] Tennant F, Hermann L (2002). Normalization of serum cortisol concentration with opioid treatment of severe chronic pain. Pain Med..

[7] Li X, Hu L (2016). The role of stress regulation on neural plasticity in pain chronification. Neural Plast..

[8] Bomholt SF, Mikkelsen JD, Blackburn-Munro G (2005). Normal hypothalamo-pituitary-adrenal axis function in a rat model of peripheral neuropathic pain. Brain Res..

[9] Ulrich-Lai YM (2006). Limbic and HPA axis function in an animal model of chronic neuropathic pain. Physiol. Behav..

[10] Yalcin I (2011). A time-dependent history of mood disorders in a murine model of neuropathic pain. Biol. Psychiatry..

[11] Riva R (2012). Comparison of the cortisolawakening response in women with shoulder and neck pain and women with fibromyalgia. Psychoneuroendocrinol..

[12] Dworkin RH, Gitlin MJ (1991). Clinical aspects of depression in chronic pain patients. Clin. J Pain..

[13] McWilliams LA, Cox BJ, Enns MW (2003). Mood and anxiety disorders associated with chronic pain: an examination in a nationally representative sample. Pain..

[14] Bushnell MC, Ceko M, Low LA (2013). Cognitive and emotional control of pain and its disruption in chronic pain. Nat. Rev. Neurosci..

[15] Neugebauer V, Li W, Bird GC, Han JS (2004). The amygdala and persistent pain. Neuroscientist..

[16] Jiang H (2014). Sensitization of neurons in the central nucleus of the amygdala via the decreased GABAergic inhibition contributes to the development of neuropathic pain-related anxiety-like behaviors in rats. Mol. Brain..

[17] Gonçalves L, Friend LV, Dickenson AH (2015). The influence of μ-opioid and noradrenaline reuptake inhibition in the modulation of pain responsive neurons in the central amygdala by tapentadol in rats with neuropathy. Eur. J. Pharmacol..

[18] Neugebauer V (2015). Amygdala pain mechanisms. Handb. Exp. Pharmacol..

[19] Neugebauer V (2007). The amygdala: different pains, different mechanisms. Pain..

[20] Thompson JM, Neugebauer V (2017). Amygdala plasticity and pain. Pain. Res. Manag..

[21] LeDoux JE (2000). Emotion circuits in the brain. Annu. Rev. Neurosci..

[22] Swanson LW, Petrovich GD (1998). What is the amygdala?. Trends Neurosci..

[23] McDonald AJ (1998). Cortical pathways to the mammalian amygdala. Prog. Neurobiol..

[24] Janak PH, Tye KM (2015). From circuits to behaviour in the amygdala. Nature..

[25] Pitkänen A, Savander V, LeDoux JE (1997). Organization of intra-amygdaloid circuitries in the rat: an emerging framework for understanding functions of the amygdala. Trends Neurosci..

[26] Ikeda R (2007). NMDA receptor-independent synaptic plasticity in the central amygdala in the rat model of neuropathic pain. Pain..

[27] Bourbia N, Ansah OB, Pertovaara A (2010). Corticotropin-releasing factor in the rat amygdala differentially influences sensory-discriminative and emotional-like pain response in peripheral neuropathy. J. Pain..

[28] Gonçalves L (2008). Neuropathic pain is associated with depressive behaviour and induces neuroplasticity in the amygdala of the rat. Exp. Neurol..

[29] Gonçalves L, Dickenson AH (2012). Asymmetric time-dependent activation of right central amygdala neurones in rats with peripheral neuropathy and pregabalin modulation. Eur. J. Neurosci..

[30] Ji G (2017). 5-HT(2C) Receptor Knockdown in the Amygdala Inhibits Neuropathic-Pain-Related Plasticity and Behaviors. J. Neurosci..

[31] Herdegen T, Leah JD (1998). Inducible and constitutive transcription factors in the mammalian nervous system: control of gene expression by Jun, Fos and Krox, and CREB/ATF proteins. Brain Res. Brain Res. Rev..

[32] Herrera DG, Robertson HA (1996). Activation of c-Fos in the brain. Prog. Neurobiol..

[33] Hunt SP, Pini A, Evan G (1987). Induction of c-fos-like protein in spinal cord neurons following sensory stimulation. Nature..

[34] McDonald AJ (1982). Cytoarchitecture of the central amygdaloid nucleus of the rat. J. Comp. Neurol..

[35] Cassell MD, Freedman LJ, Shi C (1999). The intrinsic organization of the central extended amygdala. Ann. N. Y. Acad. Sci..

[36] Jiang Y (2016). Perturbed connectivity of the amygdala and its subregions with the central executive and default mode networks in chronic pain. Pain..

[37] Carlsen J, Heimer L (1988). The basolateral amygdaloid complex as a cortical-like structure. Brain Res..

[38] Martin TJ (2011). Involvement of the lateral amygdala in the antiallodynic and reinforcing effects of heroin in rats after peripheral nerve injury. Anesthesiol..

[39] Ji G (2010). Cognitive impairment in pain through amygdala-driven prefrontal cortical deactivation. J. Neurosci..

[40] Bernard JF, Huang GF, Besson JM (1992). Nucleus centralis of the amygdala and the globus pallidus ventralis: electrophysiological evidence for an involvement in pain processes. J Neurophysiol..

[41] Carr FB, Zachariou V (2014). Nociception and pain: lessons from optogenetics. Front. Behav. Neurosci..

[42] Fu Y, Neugebauer V (2008). Differential mechanisms of CRF1 and CRF2 receptor functions in the amygdala in pain-related synaptic facilitation and behavior. J. Neurosci..

[43] Pedersen LH, Scheel-Kruger J, Blackburn-Munro G (2007). Amygdala GABA-A receptor involvement in mediating sensory-discriminative and affective-motivational pain responses in a rat model of peripheral nerve injury. Pain..

[44] Helmstetter FJ, Tershner SA, Poore LH, Bellgowan PS (1998). Antinociception following opioid stimulation of the basolateral amygdala is expressed through the periaqueductal gray and rostral ventromedial medulla. Brain Res..

[45] Shin MS, Helmstetter FJ (2005). Antinociception following application of DAMGO to the basolateral amygdala results from a direct interaction of DAMGO with Mu opioid receptors in the amygdala. Brain Res..

[46] Suzuki T (2007). Experimental neuropathy in mice is associated with delayed behavioral changes related to anxiety and depression. Anesth. Analg..

[47] Caspani O, Reitz MC, Ceci A, Kremer A, Treede RD (2014). Tramadol reduces anxiety-related and depression-associated behaviors presumably induced by pain in the chronic constriction injury model of neuropathic pain in rats. Pharmacol. Biochem. Behav..

[48] Chung G (2017). Upregulation of prefrontal metabotropic glutamate receptor 5 mediates neuropathic pain and negative mood symptoms after spinal nerve injury in rats. Sci. Rep..

[49] Zhong M (2011). Amygdala hyperactivation and prefrontal hypoactivation in subjects with cognitive vulnerability to depression. Biol Psychol..

[50] Narita M (2006). Chronic pain induces anxiety with concomitant changes in opioidergic function in the amygdala. Neuropsychopharmacol..

[51] Rubinow MJ (2016). Basolateral amygdala volume and cell numbers in major depressive disorder: a postmortem stereological study. Brain Struct. Funct..

[52] Joshi SH (2016). Structural Plasticity of the Hippocampus and Amygdala Induced by Electroconvulsive Therapy in Major Depression. Biol. Psychiatry..

[53] Chen L (2018). Activation of CRF/CRFR1 signaling in the basolateral nucleus of the amygdala contributes to chronic forced swim-induced depressive-like behaviors in rats. Behav Brain Res..

[54] Li MJ (2017). Chronic stress exacerbates neuropathic pain via the integration of stress-affect-related information with nociceptive information in the central nucleus of the amygdala. Pain..

[55] John CS (2015). Blockade of the GLT-1 Transporter in the Central Nucleus of the Amygdala Induces both Anxiety and Depressive-Like Symptoms. Neuropsychopharmacol..

[56] Hall CS (1934). Emotional behavior in the rat: I. Defecation and urination as measures of individual differences in emotionality. J. Comp. Psychol..

[57] Redolat R, Perez-Martinez A, Carrasco MC, Mesa P (2009). Individual differences in novelty-seeking and behavioral responses to nicotine: a review of animal studies. Curr. Drug Abuse Rev..

[58] Manning BH (1998). A lateralized deficit in morphine antinociception after unilateral inactivation of the central amygdala. J. Neurosci..

[59] Chen L (2016). GABA(A) Receptors in the central nucleus of the amygdala are Involved in pain- and itch-related responses. J. Pain..

[60] Seminowicz DA (2009). MRI structural brain changes associated with sensory and emotional function in a rat model of long-term neuropathic pain. Neuroimage..

[61] Filho PR (2016). Transcranial direct current stimulation (tDCS) reverts behavioral alterations and brainstem BDNF level increase induced by neuropathic pain model: Long-lasting effect. Prog. Neuropsychopharmacol. Biol. Psychiatry..

[62] Roeska K (2008). Anxiety-like behaviour in rats with mononeuropathy is reduced by the analgesic drugs morphine and gabapentin. Pain..

[63] Matsuzawa-Yanagida K (2008). Usefulness of antidepressants for improving the neuropathic pain-like state and pain-induced anxiety through actions at different brain sites. Neuropsychopharmacol..

[64] Zarrindast MR, Solati J, Oryan S, Parivar K (2008). Effect of intra-amygdala injection of nicotine and GABA receptor agents on anxiety-like behaviour in rats. Pharmacol..

[65] Moreira CM, Masson S, Carvalho MC, Brandão ML (2007). Exploratory behaviour of rats in the elevated plus-maze is differentially sensitive to inactivation of the basolateral and central amygdaloid nuclei. Brain Res. Bull..

[66] Martin JH (1991). Autoradiographic estimation of the extent of reversible inactivation produced by micro-injection of lidocaine and muscimol in the rat. Neurosci. Lett..

[67] Zimmermann M (1983). Ethical guidelines for investigations of experimental pain in conscious animals. Pain..

[68] Bennett GJ, Xie YK (1988). A peripheral mononeuropathy in rat that produces disorders of pain sensation like those seen in man. Pain..

[69] Paxinos, G. & Watson, C. The rat brain in stereotaxic coordinates - The new coronal set. Elsevier. New York: Academic Press (2005).

[70] Macedo CE, Martinez RC, Brandão ML (2006). Conditioned and unconditioned fear organized in the inferior colliculus are differentially sensitive to injections of muscimol into the basolateral nucleus of the amygdala. Behav. Neurosci..

[71] Majchrzak M, Di Scala G (2000). GABA and muscimol as reversible inactivation tools inblearning and memory. Neural Plast..

[72] Martinez RC, de Oliveira AR, Brandão ML (2006). Conditioned and unconditioned fear organized in the periaqueductal gray are differentially sensitive to injections of muscimol into amygdaloid nuclei. Neurobiol. Learn. Mem..

[73] Rea K, Roche M, Finn DP (2011). Modulation of conditioned fear, fear-conditioned analgesia, and brain regional c-Fos expression following administration of muscimol into the rat basolateral amygdala. J. Pain..

[74] Randall LO, Selitto JJ (1957). A method for measurement of analgesic activity on inflamed tissue. Arch. Int. Pharmacodyn. Ther..

[75] Milligan ED (2000). Thermal hyperalgesia and mechanical allodynia produced by intrathecal administration of the human immunodeficiency virus-1 (HIV-1) envelope glycoprotein, Gp120. Brain Res..

[76] Handley SL, Mithani S (1984). Effects of alpha-adrenoceptor agonists and antagonists in a maze-exploration model of ‘fear’-motivated behaviour. Naunyn Schmiedebergs Arch. Pharmacol..

[77] Garcia AM, Cardenas FP, Morato S (2005). Effect of different illumination levels on rat behavior in the elevated plus-maze. Physiol. Behav..

[78] Porsolt RD, Bertin A, Jalfre M (1977). Behavioral despair in mice: a primary screening test for antidepressants. Arch. Int. Pharmacodyn. Ther..

[79] Avila-Martin G (2015). Oral 2-hydroxyoleic acid inhibits reflex hypersensitivity and open-field-induced anxiety after spared nerve injury. Eur. J. Pain..

[80] Bahaaddini M (2016). The role of trigeminal nucleus caudalis orexin 1 receptor in orofacial pain-induced anxiety in rat. Neuroreport..

[81] Sergejeva M (2015). Anatomical landmarks for registration of experimental image data to volumetric rodent brain atlasing templates. J. Neurosci. Methods..

[82] Foote SL (1980). Accurate three-dimensional reconstruction of neuronal distributions in brain: reconstruction of the rat nucleus locus coeruleus. J. Neurosci. Methods..

[83] Martinez RC (2013). Active vs. reactive threat responding is associated with differential c-Fos expression in specific regions of amygdala and prefrontal cortex. Learn. Mem..

