# Supplementary material for: The critical role of amygdala subnuclei in nociceptive and depressive-like behaviors in peripheral neuropathy
Source: Sci Rep. 2018 Sep 11;8:13608. doi: 10.1038/s41598-018-31962-w (PMC6134132; doi:10.1038/s41598-018-31962-w)
Supplement: Supplementary file 1 — Supplementary information [file 41598_2018_31962_MOESM1_ESM.docx]

**Supplementary info**

**The critical role of amygdala subnuclei in nociceptive and depressive-like behaviors in peripheral neuropathy**

Midiã D. J. Seno+, Danielle V. Assis+, Flávia V. Gouveia+; Geiza F. Antunes, Mayra A. Kuroki; Caroline C. Oliveira; Lennon C. T. Santos; Rosana L. Pagano&, and Raquel C. R. Martinez&*

Running head: Amygdala nuclei and neuropathic pain

Highlights:

The basolateral and central amygdala are critical for chronic pain

Understanding the roles of amygdala nuclei in neuropathic pain

Amygdala nuclei affect the nociceptive response and depression

Keywords: neuropathic pain; amygdala; anxiety; depression; adrenocorticotrophic hormone; corticosterone; HPA axis

**Supplementary Figure S1.** Representative photomicrographs shown along Bregma. Transverse c-Fos-IR sections illustrating labeling in the basolateral (BLA), lateral (LA) and central (CeA) nuclei of the amygdala in the naive group (**A**, **B**, **C**), sham group (**D**, **E**, **F**), and animals submitted to chronic constriction injury (CCI) of the sciatic nerve (**G**, **H**, **I**). Transverse adjoining Nissl-stained sections used to define amygdala subnuclei based on histological landmarks along different bregma levels (**J, K, L**). Magnification: 10X. Scale bars=200 μm. IR: immunoreactivity.


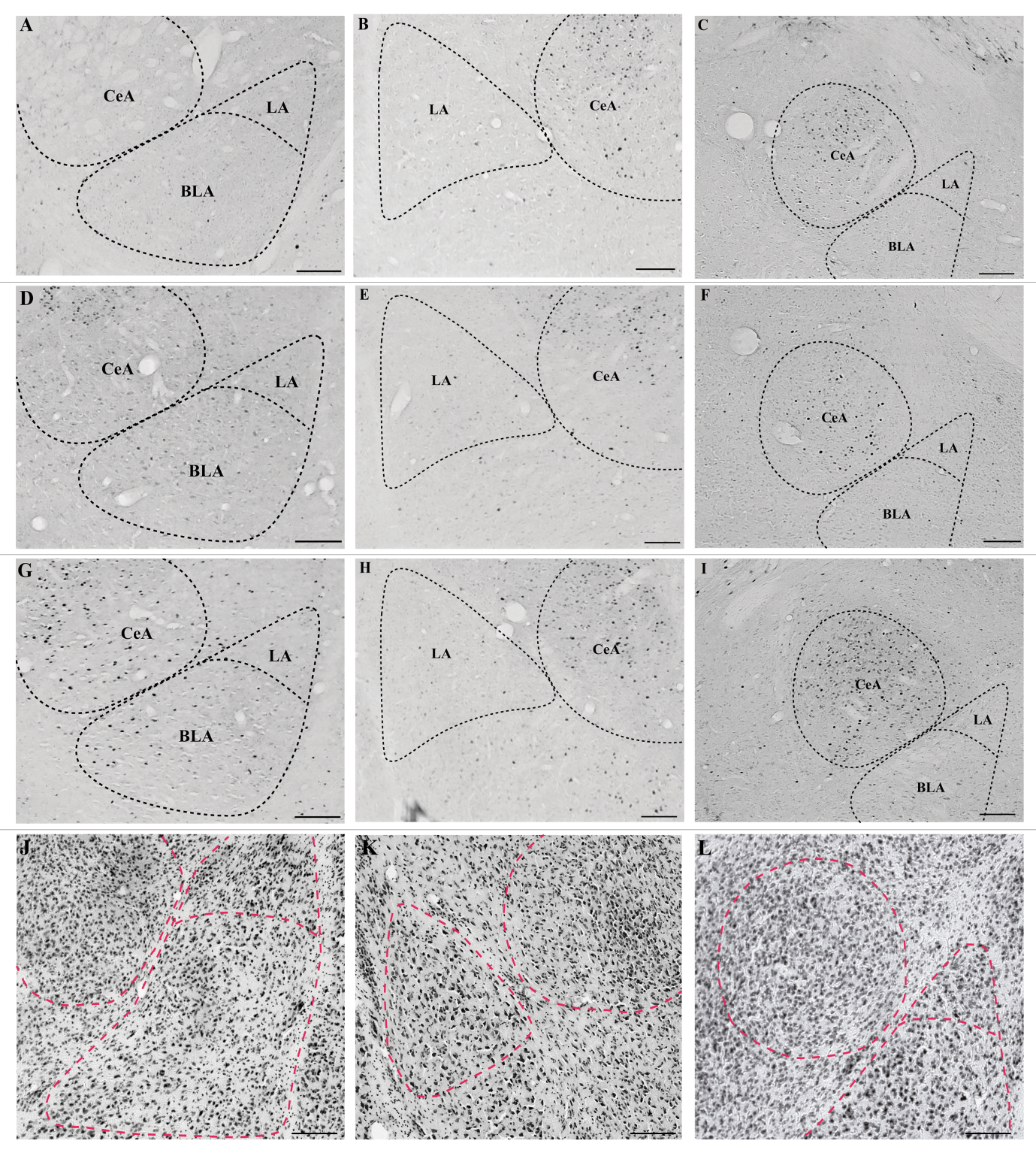


**Supplementary Figure S2.** Evaluation of mechanical hyperalgesia in the intact paw (left paw) of naive rats, sham-operated rats (Sham), and animals submitted to chronic constriction injury (CCI) of the sciatic nerve during basal measurements (M1 - black), measurement 2 (M2 – light gray) and measurements taken after the microinjection (M3 – dark gray) of saline or muscimol targeted to the basolateral nucleus (**A**) or central nucleus (**B**) of the amygdala.

| 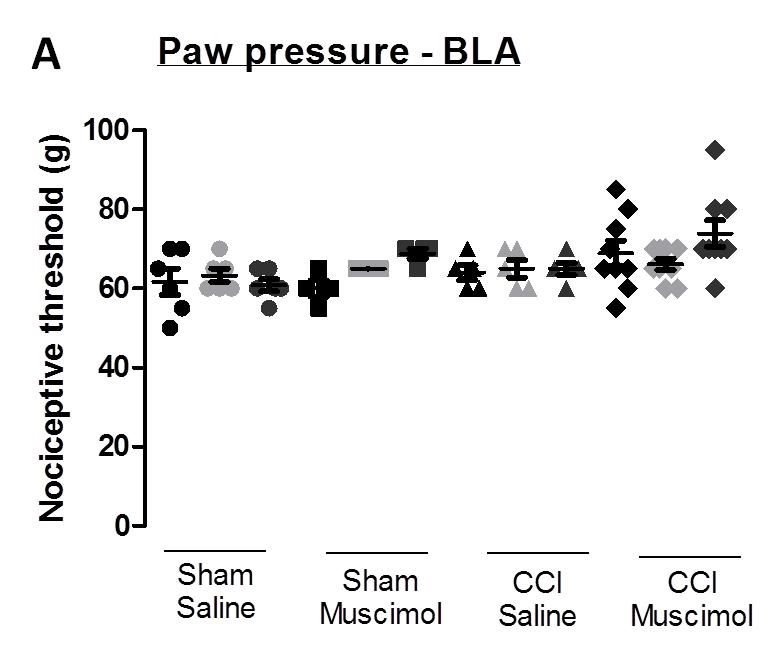 | 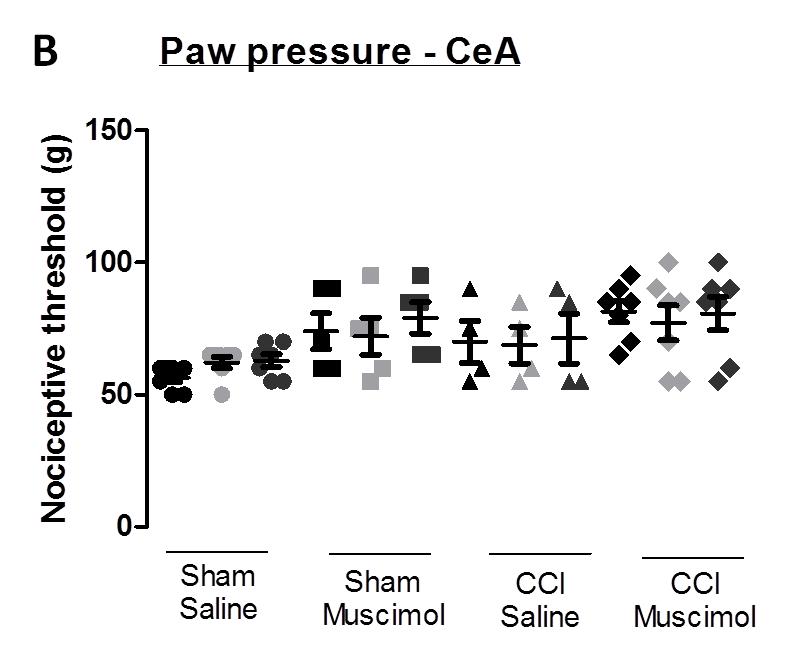 |
| --- | --- |

**Supplementary Table 1**. The spatial and ethological parameters exhibited in the open field test by sham-operated rats (Sham) and animals submitted to chronic constriction injury (CCI) of the sciatic nerve after the microinjection of saline or muscimol targeted to the basolateral (BLA) or central (CeA) nucleus of the amygdala. Values are shown as the mean ± standard error of the mean (Mean ± SEM). Two-way ANOVA was used to test all behavioral measurements.

| **Parameters** | | **Sham Saline** | | **Sham Muscimol** | | **CCI**  **Saline** | | **CCI Muscimol** | | **F_(1,19)_; p** | |
| --- | --- | --- | --- | --- | --- | --- | --- | --- | --- | --- | --- |
| **BLA - Displacement Measures** | | | | | | | | | | | |
| Entries in the periphery area | | 84.2 ± 5.10 | | 69.3 ± 5.2 | | 61.8 ± 3.4 | | 68.0 ± 8.6 | | Factor 1: F=2.29; p=0.14  Factor 2: F=0.31; p=0.58  Interaction F= 1.83; p=0.19 | |
| Entries in the central area | | 6.2 ± 1.71 | | 6.8 ± 2.13 | | 4.0 ± 0.94 | | 4.4 ± 1.01 | | Factor 1: F=1.51; p=0.23  Factor 2: F=0.06; p=0.79  Interaction F= 0.00; p=0.95 | |
| Total entries | | 90.3 ± 3.5 | | 76.0 ± 4.3 | | 65.8 ± 2.1 | | 72.4 ± 5.5 | | Factor 1: F=2.46; p=0.13  Factor 2: F=0.18; p=0.67  Interaction F= 1.35; p=0.25 | |
| Time spent in the periphery | | 288.3 ± 2.1 | | 285.1 ± 3.5 | | 294.9 ± 0.9 | | 289.8 ± 2.0 | | Factor 1: F=1.53; p=0.23  Factor 2: F=0.83; p=0.37  Interaction F= 0.04; p=0.83 | |
| Time spent in central area | | 11.7 ± 2.1 | | 14.9 ± 3.5 | | 5.1 ± 0.9 | | 10.2 ± 2.0 | | Factor 1: F=1.53; p=0.23  Factor 2: F=0.83; p=0.37  Interaction F= 0.04; p=0.83 | |
| Total distance in the periphery area | | 841.7 ± 40.4 | | 692.5 ± 41.4 | | 618.0 ± 27.1 | | 680.0 ± 67.4 | | Factor 1: F=2.29; p=0.14  Factor 2: F=0.31; p=0.58  Interaction F= 1.83; p=0.19 | |
| Total distance in the central area | | 61.7 ± 10.4 | | 67.5 ± 13.1 | | 40.0 ± 5.8 | | 43.8 ± 6.2 | | Factor 1: F=1.51; p=0.23  Factor 2: F=0.06; p=0.79  Interaction F= 0.00; p=0.95 | |
| Total distance | | 903.3 ± 34.6 | | 760.0 ± 43.3 | | 658.0 ± 21.5 | | 723.8 ± 54.6 | | Factor 1: F=2.46; p=0.13  Factor 2: F=0.18; p=0.67  Interaction F= 1.35; p=0.25 | |
| **BLA - Ethological Measures** | | | | | | | | | | | |
| Rearing in the periphery | | 0.2 ± 0.1 | | 1.8 ± 0.7 | | 1.8 ± 0.6 | | 4.0 ± 1.2 | | Factor 1: F=1.16; p=0.29  Factor 2: F=1.10; p=0.30  Interaction F= 0.02; p=0.86 | |
| Time spent in the periphery area doing rearing | | 0.0 ± 0.0 | | 0.6 ± 0.2 | | 0.5 ± 0.2 | | 1.1 ± 0.3 | | Factor 1: F=1.07; p=0.31  Factor 2: F=1.15; p=0.29  Interaction F= 0.00; p=0.99 | |
| Grooming in the periphery | | 18.0 ± 2.2 | | 16.0 ± 1.2 | | 17.6 ± 1.0 | | 17.9 ± 1.6 | | Factor 1: F=0.04; p=0.83  Factor 2: F=0.06; p=0.80  Interaction F= 0.10; p=0.74 | |
| Total grooming | | 18.5 ± 2.3 | | 16.0 ± 1.2 | | 17.6 ± 1.0 | | 18.1 ± 1.8 | | Factor 1: F=0.02; p=0.86  Factor 2: F=0.07; p=0.78  Interaction F= 0.17; p=0.68 | |
| Time spent in the periphery doing grooming | | 4.6 ± 0.7 | | 4.7 ± 0.5 | | 4.4 ± 0.2 | | 4.6 ± 0.4 | | Factor 1: F=0.02; p=0.87  Factor 2: F=0.03; p=0.85  Interaction F= 0.00; p=0.97 | |
| Total time spent in grooming | | 4.8 ± 0.7 | | 4.7 ± 0.5 | | 4.7 ± 0.5 | | 4.7 ± 0.5 | | Factor 1: F=0.00; p=0.99  Factor 2: F=0.00; p=0.99  Interaction F= 0.00; p=0.99 | |
| **CeA - Displacement Measures** | | | | | | | | | | |  |
| Entries in the periphery area | 84.2 ± 5.10 | | 69.3 ± 5.2 | | 61.8 ± 3.4 | | 68.0 ± 8.6 | | Factor 1: F=2.29; p=0.14  Factor 2: F=0.31; p=0.58  Interaction F= 1.83; p=0.19 | |  |
| Entries in the central area | 6.2 ± 1.71 | | 6.8 ± 2.13 | | 4.0 ± 0.94 | | 4.4 ± 1.01 | | Factor 1: F=1.51; p=0.23  Factor 2: F=0.06; p=0.79  Interaction F= 0.00; p=0.95 | |  |
| Total entries | 90.3 ± 3.5 | | 76.0 ± 4.3 | | 65.8 ± 2.1 | | 72.4 ± 5.5 | | Factor 1: F=2.46; p=0.13  Factor 2: F=0.18; p=0.67  Interaction F= 1.35; p=0.25 | |  |
| Time spent in the periphery | 288.3 ± 2.1 | | 285.1 ± 3.5 | | 294.9 ± 0.9 | | 289.8 ± 2.0 | | Factor 1: F=1.53; p=0.23  Factor 2: F=0.83; p=0.37  Interaction F= 0.04; p=0.83 | |  |
| Time spent in central area | 11.7 ± 2.1 | | 14.9 ± 3.5 | | 5.1 ± 0.9 | | 10.2 ± 2.0 | | Factor 1: F=1.53; p=0.23  Factor 2: F=0.83; p=0.37  Interaction F= 0.04; p=0.83 | |  |
| Total distance in the periphery area | 841.7 ± 40.4 | | 692.5 ± 41.4 | | 618.0 ± 27.1 | | 680.0 ± 67.4 | | Factor 1: F=2.29; p=0.14  Factor 2: F=0.31; p=0.58  Interaction F= 1.83; p=0.19 | |  |
| Total distance in the central area | 61.7 ± 10.4 | | 67.5 ± 13.1 | | 40.0 ± 5.8 | | 43.8 ± 6.2 | | Factor 1: F=1.51; p=0.23  Factor 2: F=0.06; p=0.79  Interaction F= 0.00; p=0.95 | |  |
| Total distance | 903.3 ± 34.6 | | 760.0 ± 43.3 | | 658.0 ± 21.5 | | 723.8 ± 54.6 | | Factor 1: F=2.46; p=0.13  Factor 2: F=0.18; p=0.67  Interaction F= 1.35; p=0.25 | |  |
| **CeA - Ethological Measures** | | | | | | | | | | |  |
| Rearing in the periphery | 0.2 ± 0.1 | | 1.8 ± 0.7 | | 1.8 ± 0.6 | | 4.0 ± 1.2 | | Factor 1: F=1.16; p=0.29  Factor 2: F=1.10; p=0.30  Interaction F= 0.02; p=0.86 | |  |
| Time spent in the periphery area doing rearing | 0.0 ± 0.0 | | 0.6 ± 0.2 | | 0.5 ± 0.2 | | 1.1 ± 0.3 | | Factor 1: F=1.07; p=0.31  Factor 2: F=1.15; p=0.29  Interaction F= 0.00; p=0.99 | |  |
| Grooming in the periphery | 18.0 ± 2.2 | | 16.0 ± 1.2 | | 17.6 ± 1.0 | | 17.9 ± 1.6 | | Factor 1: F=0.04; p=0.83  Factor 2: F=0.06; p=0.80  Interaction F= 0.10; p=0.74 | |  |
| Total grooming | 18.5 ± 2.3 | | 16.0 ± 1.2 | | 17.6 ± 1.0 | | 18.1 ± 1.8 | | Factor 1: F=0.02; p=0.86  Factor 2: F=0.07; p=0.78  Interaction F= 0.17; p=0.68 | |  |
| Time spent in the periphery doing grooming | 4.6 ± 0.7 | | 4.7 ± 0.5 | | 4.4 ± 0.2 | | 4.6 ± 0.4 | | Factor 1: F=0.02; p=0.87  Factor 2: F=0.03; p=0.85  Interaction F= 0.00; p=0.97 | |  |
| Total time spent in grooming | 4.8 ± 0.7 | | 4.7 ± 0.5 | | 4.7 ± 0.5 | | 4.7 ± 0.5 | | Factor 1: F=0.00; p=0.99  Factor 2: F=0.00; p=0.99  Interaction F= 0.00; p=0.99 | |  |

**Supplementary Table 2**. Spatial and ethological parameters exhibited in the elevated plus-maze by sham-operated rats (Sham) and animals submitted to chronic constriction injury (CCI) of the sciatic nerve after the microinjection of saline or muscimol targeted to the basolateral (BLA) or central (CeA) nucleus of the amygdala. Values are shown as the mean ± standard error of the mean (Mean ± SEM). Two-way ANOVA was used for all behavioral measurements.

| **Parameters** | | **Sham Saline** | | **Sham Muscimol** | | **CCI**  **Saline** | | | **CCI Muscimol** | | **F_(1,19)_; p** | | |
| --- | --- | --- | --- | --- | --- | --- | --- | --- | --- | --- | --- | --- | --- |
| **BLA - Displacement Measures** | | | | | | | | | | | | | |
| Entries into the closed arms | | 6.8 ± 0.53 | | 5.8 ± 0.32 | | 6.8 ± 0.55 | | | 8.3 ± 1.29 | | Factor 1: F=1.18; p=0.29  Factor 2: F=0.02; p=0.87  Interaction F= 1.25; p=0.27 | | |
| % Entries into the closed arms | | 54.7 ± 3.4 | | 55.0 ± 3.8 | | 42.9 ± 2.7 | | | 50.1 ± 2.1 | | Factor 1: F=4.45; p=0.04  Factor 2: F=0.89; p=0.35  Interaction F= 0.73; p=0.40 | | |
| Entries into the closed arms extremities | | 5.3 ± 0.7 | | 4.8 ± 0.1 | | 6.0 ± 0.4 | | | 3.8 ± 0.7 | | Factor 1: F=0.02; p=0.88  Factor 2: F=1.56; p=0.22  Interaction F= 0.54; p=0.47 | | |
| Time spent closed arms | | 108.5 ± 11.5 | | 108.6 ± 3.5 | | 139.4 ± 11.7 | | | 111.1 ± 14.7 | | Factor 1: F=0.49; p=0.49  Factor 2: F=0.35; p=0.55  Interaction F= 0.35; p=0.55 | | |
| Time spent closed arms extremities | | 40.7 ± 5.8 | | 44.3 ± 0.7 | | 79.2 ± 6.7 | | | 45.4 ± 8.5 | | Factor 1: F=2.36; p=0.14  Factor 2: F=1.37; p=0.25  Interaction F= 2.10; p=0.16 | | |
| Time spent central area | | 41.5 ± 2.3 | | 73.3 ± 1.8 | | 60.5 ± 0.5 | | | 62.2 ± 2.3 | | Factor 1: F=0.41; p=0.52  Factor 2: F=7.63; p=0.01  Interaction F= 6.15; p=0.02 | | |
| **BLA - Ethological Measures** | | | | | | | | | | | | | |
| Dipping in the open arms | | 8.0 ± 2.4 | | 3.0 ± 0.3 | | 3.0 ± 0.6 | | 7.6 ± 2.0 | | | | Factor 1: F=0.00; p=0.95  Factor 2: F=0.00; p=0.95  Interaction F= 2.13; p=0.16 | |
| Dipping in the closed arms | | 10.0 ± 1.4 | | 7.3 ± 0.9 | | 10.4 ± 1.0 | | 6.5 ± 1.5 | | | | Factor 1: F=0.00; p=0.94  Factor 2: F=1.91; p=0.18  Interaction F= 0.05; p=0.81 | |
| Dipping in the open arms extremities | | 4.2 ± 1.5 | | 1.8 ± 0.1 | | 1.8 ± 0.5 | | 5.0 ± 1.4 | | | | Factor 1: F=0.03; p=0.84  Factor 2: F=0.03; p=0.86  Interaction F= 1.59; p=0.22 | |
| Dipping in the closed arms extremities | | 5.5 ± 0.5 | | 5.0 ± 0.2 | | 8.6 ± 0.5 | | 4.6± 0.6 | | | | Factor 1: F=0.75; p=0.39  Factor 2: F=2.04; p=0.16  Interaction F= 1.23; p=0.28 | |
| Dipping in the central area | | 0.5 ± 0.1 | | 1.0 ± 0.2 | | 0.6 ± 0.1 | | 1.1± 0.2 | | | | Factor 1: F=0.06; p=0.80  Factor 2: F=1.24; p=0.27  Interaction F= 0.00; p=0.97 | |
| Dipping in the open arms | | 8.0 ± 2.4 | | 3.0 ± 0.3 | | 3.0 ± 0.6 | | 7.6± 2.0 | | | | Factor 1: F=0.00; p=0.95  Factor 2: F=0.00; p=0.95  Interaction F= 2.13; p=0.16 | |
| Dipping in the closed arms | | 10.0 ± 1.4 | | 7.3 ± 0.9 | | 10.4 ± 1.0 | | 6.5± 1.5 | | | | Factor 1: F=0.00; p=0.94  Factor 2: F=1.91; p=0.18  Interaction F= 0.05; p=0.81 | |
| Dipping into the open arms extremities | | 4.2 ± 1.5 | | 1.8 ± 0.1 | | 1.8 ± 0.5 | | 5.0± 1.4 | | | | Factor 1: F=0.03; p=0.84  Factor 2: F=0.03; p=0.86  Interaction F= 1.59; p=0.22 | |
| Dipping into the closed arms extremities | | 5.5 ± 0.5 | | 5.0 ± 0.2 | | 8.6 ± 0.5 | | 4.6± 0.6 | | | | Factor 1: F=0.75; p=0.39  Factor 2: F=2.04; p=0.16  Interaction F= 1.23; p=0.28 | |
| Time spent dipping - open arms | | 2.8 ± 0.5 | | 0.9 ± 0.1 | | 1.0 ± 0.1 | | 2.8± 0.5 | | | | Factor 1: F=0.00; p=0.96  Factor 2: F=0.00; p=0.99  Interaction F= 2.38; p=0.13 | |
| Time spent dipping - closed arms | | 3.6 ± 0.3 | | 2.4 ± 0.2 | | 4.4 ± 0.4 | | 2.8± 0.5 | | | | Factor 1: F=0.27; p=0.60  Factor 2: F=1.50; p=0.23  Interaction F= 0.02; p=0.88 | |
| Time spent dipping - open arms extremities | | 1.5 ± 0.3 | | 0.4 ± 0.0 | | 0.6 ± 0.1 | | 1.8± 0.3 | | | | Factor 1: F=0.10; p=0.74  Factor 2: F=0.00; p=0.93  Interaction F= 1.91; p=0.18 | |
| Time spent dipping -closed arms extremities | | 2.0 ± 0.2 | | 1.6 ± 0.1 | | 3.7 ± 0.3 | | 2.2± 0.4 | | | | Factor 1: F=1.51; p=0.23  Factor 2: F=1.06; p=0.31  Interaction F= 0.38; p=0.54 | |
| Time spent dipping -central area | | 0.1± 0.0 | | 0.4 ± 0.1 | | 0.2 ± 0.0 | | 0.4± 0.1 | | | | Factor 1: F=0.10; p=0.75  Factor 2: F=1.47; p=0.24  Interaction F= 0.00; p=0.98 | |
| Grooming in the open arms | | 13.0± 0.5 | | 11.0 ± 0.6 | | 7.6 ± 0.6 | | 13.3± 1.2 | | | | Factor 1: F=0.35; p=0.55  Factor 2: F=0.47; p=0.49  Interaction F= 2.08; p=0.16 | |
| Grooming in the closed arms | | 9.3± 0.6 | | 10.0 ± 0.5 | | 8.4 ± 0.6 | | 10.9± 0.4 | | | | Factor 1: F=0.00; p=0.98  Factor 2: F=0.94; p=0.34  Interaction F= 0.31; p=0.58 | |
| Grooming in the open arms extremities | | 3.2± 0.2 | | 2.0 ± 0.1 | | 2.2 ± 0.3 | | 5.3± 0.8 | | | | Factor 1: F=0.48; p=0.49  Factor 2: F=0.33; p=0.57  Interaction F= 1.65; p=0.21 | |
| Grooming in the closed arms extremities | | 4.7± 0.4 | | 4.0 ± 0.1 | | 6.4 ± 0.3 | | 4.6± 0.5 | | | | Factor 1: F=0.96; p=0.33  Factor 2: F=1.02; p=0.32  Interaction F= 0.21; p=0.65 | |
| Grooming in the central area | | 3.8± 0.5 | | 4.3 ± 0.1 | | 1.2 ± 0.2 | | 4.5± 0.3 | | | | Factor 1: F=1.68; p=0.20  Factor 2: F=4.09; p=0.05  Interaction F= 2.46; p=0.13 | |
| Total grooming | | 26.2± 0.9 | | 25.3 ± 0.9 | | 17.2 ± 0.4 | | 28.6± 1.4 | | | | Factor 1: F=0.73; p=0.40  Factor 2: F=2.58; p=0.12  Interaction F= 3.57; p=0.07 | |
| Time spent grooming - open arms | | 3.5± 0.2 | | 2.4 ± 0.2 | | 2.3 ± 0.2 | | 3.6± 0.3 | | | | Factor 1: F=0.00; p=0.97  Factor 2: F=0.03; p=0.84  Interaction F= 2.41; p=0.13 | |
| Time spent grooming – closed arms | | 2.3± 0.2 | | 2.1 ± 0.2 | | 2.4 ± 0.2 | | 3.0± 0.1 | | | | Factor 1: F=1.16; p=0.29  Factor 2: F=0.08; p=0.77  Interaction F= 0.63; p=0.43 | |
| Time spent grooming – central area | | 1.0± 0.1 | | 1.0 ± 0.0 | | 0.3 ± 0.0 | | 1.3± 0.1 | | | | Factor 1: F=0.37; p=0.54  Factor 2: F=3.83; p=0.06  Interaction F= 3.45; p=0.07 | |
| Total time spent in grooming | | 6.7± 0.4 | | 5.5 ± 0.3 | | 5.0 ± 0.1 | | 7.8± 0.3 | | | | Factor 1: F=0.11; p=0.73  Factor 2: F=0.61; p=0.44  Interaction F= 4.27; p=0.05 | |
| Time spent in freezing – open arms | | 3.5± 0.5 | | 4.5 ± 0.4 | | 3.0 ± 0.2 | | 2.1± 0.3 | | | | Factor 1: F=1.17; p=0.20  Factor 2: F=0.00; p=0.95  Interaction F= 0.74; p=0.39 | |
| Total freezing | | 4.2± 0.5 | | 5.0 ± 0.5 | | 3.0 ± 0.2 | | 2.5± 0.3 | | | | Factor 1: F=2.67; p=0.11  Factor 2: F=0.02; p=0.88  Interaction F= 0.35; p=0.55 | |
| Freezing in the open arms | | 1.2± 0.2 | | 1.6 ± 0.1 | | 1.1 ± 0.1 | | 0.8± 0.1 | | | | Factor 1: F=1.27; p=0.27  Factor 2: F=0.03; p=0.85  Interaction F= 0.82; p=0.37 | |
| Total time of freezing | | 1.4± 0.2 | | 1.7 ± 0.1 | | 1.1 ± 0.1 | | 1.1± 0.1 | | | | Factor 1: F=1.64; p=0.21  Factor 2: F=0.09; p=0.75  Interaction F= 0.16; p=0.69 | |
| Stretching in the open arms | | 3.2± 0.3 | | 2.3 ± 0.2 | | 2.2 ± 0.2 | | 1.4± 0.3 | | | | Factor 1: F=1.57; p=0.22  Factor 2: F=1.31; p=0.26  Interaction F= 0.00; p=0.99 | |
| Stretching in the open arms extremities | | 1.2± 0.2 | | 0.4 ± 0.0 | | 0.8 ± 0.1 | | 0.5± 0.1 | | | | Factor 1: F=0.14; p=0.71  Factor 2: F=1.80; p=0.19  Interaction F= 0.23; p=0.63 | |
| **CeA - Displacement Measures** | | | | | | | | | | | | |  |
| Entries into the closed arms | 6.4 ± 0.23 | | 5.4 ± 0.40 | | 8.2 ± 1.07 | | 6.6 ± 0.63 | | | Factor 1: F=2.12; p=0.15  Factor 2: F=1.59; p=0.21  Interaction F= 0.07; p=0.78 | | |  |
| % Entries into the closed arms | 53.5 ± 1.6 | | 51.6 ± 2.6 | | 56.4 ± 3.5 | | 51.0 ± 1.9 | | | Factor 1: F=0.09; p=0.76  Factor 2: F=0.85; p=0.36  Interaction F= 0.17; p=0.68 | | |  |
| Entries into the closed arms extremities | 3.6 ± 0.3 | | 1.6 ± 0.4 | | 3.8 ± 0.9 | | 2.3 ± 0.7 | | | Factor 1: F=0.21; p=0.64  Factor 2: F=3.25; p=0.08  Interaction F= 0.05; p=0.82 | | |  |
| Time spent closed arms | 113.9 ± 12.2 | | 63.2 ± 9.5 | | 90.0 ± 14.3 | | 54.7 ± 8.0 | | | Factor 1: F=0.68; p=0.41  Factor 2: F=4.93; p=0.03  Interaction F= 0.15; p=0.69 | | |  |
| Time spent closed arms extremities | 30.4 ± 3.4 | | 20.0 ± 6.6 | | 24.7 ± 5.8 | | 20.0 ± 5.3 | | | Factor 1: F=0.09; p=0.76  Factor 2: F=0.65; p=0.42  Interaction F= 0.09; p=0.76 | | |  |
| Time spent central area | 70.4 ± 7.6 | | 45.1 ± 5.2 | | 66.2 ± 4.5 | | 44.5 ± 6.0 | | | Factor 1: F=0.05; p=0.82  Factor 2: F=4.72; p=0.04  Interaction F= 0.02; p=0.86 | | |  |
| **CeA - Ethological Measures** | | | | | | | | | | | | |  |
| Dipping in the open arms | 2.1 ± 0.4 | | 9.7 ± 1.2 | | 8.8 ± 1.7 | | 9.6 ± 1.5 | | | Factor 1: F=2.21; p=0.15  Factor 2: F=3.61; p=0.06  Interaction F= 2.33; p=0.14 | | |  |
| Dipping in the closed arms | 10.9 ± 0.8 | | 3.0 ± 0.7 | | 6.6 ± 11.5 | | 3.6 ± 1.1 | | | Factor 1: F=1.05; p=0.31  Factor 2: F=9.40; p=0.005  Interaction F= 1.91; p=0.18 | | |  |
| Dipping in the open arms extremities | 0.6 ± 0.2 | | 4.7 ± 0.8 | | 4.6 ± 1.2 | | 4.8 ± 0.7 | | | Factor 1: F=2.35; p=0.13  Factor 2: F=2.63; p=0.11  Interaction F= 2.27; p=0.14 | | |  |
| Dipping in the closed arms extremities | 5.4 ± 0.6 | | 1.6 ± 0.6 | | 3.2 ± 0.8 | | 2.4± 0.8 | | | Factor 1: F=0.32; p=0.57  Factor 2: F=3.52; p=0.07  Interaction F= 1.47; p=0.23 | | |  |
| Dipping in the central area | 1.4 ± 0.3 | | 2.0 ± 0.4 | | 2.4 ± 0.3 | | 1.9± 0.5 | | | Factor 1: F=0.32; p=0.57  Factor 2: F=0.00; p=0.97  Interaction F= 0.55; p=0.46 | | |  |
| Time spent dipping - open arms | 0.7 ± 0.1 | | 4.1 ± 0.6 | | 3.2 ± 0.6 | | 3.4± 0.5 | | | Factor 1: F=1.21; p=0.28  Factor 2: F=4.30; p=0.04  Interaction F= 3.35; p=0.07 | | |  |
| Time spent dipping - closed arms | 3.7 ± 0.3 | | 1.0 ± 0.2 | | 2.3 ± 0.6 | | 1.4± 0.4 | | | Factor 1: F=0.55; p=0.46  Factor 2: F=7.43; p=0.01*  Interaction F= 1.67; p=0.20 | | |  |
| Time spent dipping - open arms extremities | 0.2 ± 0.1 | | 2.1 ± 0.4 | | 1.8 ± 0.5 | | 1.8± 0.3 | | | Factor 1: F=1.30; p=0.26  Factor 2: F=2.79; p=0.10  Interaction F= 2.96; p=0.09 | | |  |
| Time spent dipping -closed arms extremities | 1.8 ± 0.2 | | 0.6 ± 0.2 | | 1.2 ± 0.3 | | 1.0± 0.3 | | | Factor 1: F=0.05; p=0.81  Factor 2: F=2.45; p=0.13  Interaction F= 1.23; p=0.27 | | |  |
| Time spent dipping -central area | 0.7 ± 0.2 | | 0.7 ± 0.2 | | 0.8 ± 0.1 | | 0.6± 0.2 | | | Factor 1: F=0.01; p=0.91  Factor 2: F=0.03; p=0.85  Interaction F= 0.04; p=0.82 | | |  |
| Rearing in the open arms | 0.3 ± 0.1 | | 2.1 ± 0.6 | | 0.2 ± 0.1 | | 2.0± 0.7 | | | Factor 1: F=0.01; p=0.89  Factor 2: F=4.45; p=0.04  Interaction F= 0.00; p=0.97 | | |  |
| Rearing in the closed arms | 0.6± 0.3 | | 0.1 ± 0.1 | | 1.2 ± 0.6 | | 0.5± 0.3 | | | Factor 1: F=0.63; p=0.43  Factor 2: F=0.83; p=0.37  Interaction F= 0.04; p=0.82 | | |  |
| Rearing in the open arms extremities | 0.1 ± 0.1 | | 1.6 ± 0.6 | | 0.2 ± 0.1 | | 1.4± 0.7 | | | Factor 1: F=0.00; p=0.93  Factor 2: F=2.29; p=0.14  Interaction F= 0.02; p=0.88 | | |  |
| Time spent rearing – open arms | 0.1 ± 0.0 | | 0.7± 0.2 | | 0.0 ± 0.0 | | 0.5± 0.2 | | | Factor 1: F=0.09; p=0.75  Factor 2: F=4.97; p=0.03  Interaction F= 0.06; p=0.80 | | |  |
| Time spent rearing – closed arms | 00.1 ± 0.1 | | 0.1 ± 0.0 | | 0.4 ± 0.2 | | 0.1± 0.1 | | | Factor 1: F=0.67; p=0.42  Factor 2: F=0.86; p=0.36  Interaction F= 0.18; p=0.67 | | |  |
| Time spent rearing – open arms extremities | 0.0 ± 0.0 | | 0.5 ± 0.2 | | 0.0 ± 0.0 | | 0.4± 0.2 | | | Factor 1: F=0.01; p=0.90  Factor 2: F=2.49; p=0.128  Interaction F= 0.03; p=0.86 | | |  |
| Grooming in the open arms | 15.0 ± 1.3 | | 17.3 ± 1.0 | | 17.6 ± 1.9 | | 1.3± 1.0 | | | Factor 1: F=0.14; p=0.70  Factor 2: F=0.27; p=0.60  Interaction F= 2.41; p=0.13 | | |  |
| Grooming in the closed arms | 11.1 ± 1.1 | | 7.9 ± 1.5 | | 9.4± 1.1 | | 6.8± 1.1 | | | Factor 1: F=0.46; p=0.50  Factor 2: F=2.02; p=0.16  Interaction F= 0.02; p=0.88 | | |  |
| Grooming in the open arms extremities | 3.3± 0.6 | | 5.7 ± 0.8 | | 6.0 ± 1.3 | | 4.8± 0.5 | | | Factor 1: F=0.41; p=0.52  Factor 2: F=0.18; p=0.66  Interaction F= 1.81; p=0.19 | | |  |
| Grooming in the closed arms extremities | 4.1± 0.8 | | 2.0 ± 0.9 | | 3.2 ± 0.8 | | 2.3± 0.6 | | | Factor 1: F=0.07; p=0.79  Factor 2: F=1.40; p=0.24  Interaction F= 0.20; p=0.65 | | |  |
| Grooming in the central area | 4.1± 0.5 | | 5.3 ± 0.8 | | 5.0 ± 0.3 | | 2.8± 0.5 | | | Factor 1: F=0.71; p=0.40  Factor 2: F=0.31; p=0.58  Interaction F= 2.93; p=0.10 | | |  |
| Total grooming | 30.3± 1.0 | | 30.4 ± 2.4 | | 32.0 ± 1.1 | | 22.5± 1.7 | | | Factor 1: F=1.11; p=0.30  Factor 2: F=2.53; p=0.12  Interaction F= 2.69; p=0.11 | | |  |
| Time spent grooming - open arms | 3.9± 0.3 | | 4.5 ± 0.3 | | 4.5 ± 0.5 | | 3.4± 0.2 | | | Factor 1: F=0.16; p=0.68  Factor 2: F=0.19; p=0.66  Interaction F= 1.91; p=0.18 | | |  |
| Time spent grooming – closed arms | 2.8± 0.5 | | 2.0 ± 0.4 | | 2.2 ± 0.2 | | 1.6± 0.3 | | | Factor 1: F=0.87; p=0.35  Factor 2: F=1.41; p=0.24  Interaction F= 0.05; p=0.81 | | |  |
| Time spent grooming – central area | 1.1± 0.2 | | 1.3 ± 0.2 | | 1.3 ± 0.1 | | 0.7± 0.1 | | | Factor 1: F=0.76; p=0.38  Factor 2: F=0.44; p=0.51  Interaction F= 2.21; p=015 | | |  |
| Total time spent in grooming | 7.8± 0.3 | | 7.8 ± 0.7 | | 7.9 ± 0.3 | | 5.8± 0.4 | | | Factor 1: F=5.23; p=0.30  Factor 2: F=0.02; p=0.87  Interaction F= 0.02; p=0.88 | | |  |
| Time spent in freezing – open arms | 2.7± 0.4 | | 0.7 ± 0.2 | | 2.4 ± 0.3 | | 1.4± 0.3 | | | Factor 1: F=0.03; p=0.84  Factor 2: F=2.87; p=0.10  Interaction F= 0.29; p=0.59 | | |  |
| Freezing in the central area | 0.7± 0.2 | | 0.1 ± 0.1 | | 0.2 ± 0.1 | | 0.3± 0.1 | | | Factor 1: F=0.49; p=0.48  Factor 2: F=0.81; p=0.37  Interaction F= 1.15; p=0.29 | | |  |
| Total freezing | 3.6± 0.5 | | 2.0 ± 0.3 | | 2.8 ± 0.4 | | 1.6± 0.3 | | | Factor 1: F=0.30; p=0.58  Factor 2: F=1.72; p=0.20  Interaction F= 0.03; p=0.85 | | |  |
| Freezing no aberto | 0.9± 0.1 | | 0.3 ± 0.1 | | 0.7 ± 0.1 | | 0.5± 0.1 | | | Factor 1: F=0.00; p=0.96  Factor 2: F=2.27; p=0.14  Interaction F= 0.29; p=0.59 | | |  |
| Freezing in the central area | 0.2± 0.1 | | 0.1 ± 0.0 | | 0.1 ± 0.0 | | 0.1± 0.0 | | | Factor 1: F=0.78; p=0.38  Factor 2: F=1.04; p=0.31  Interaction F= 0.94; p=0.34 | | |  |
| Total time of freezing | 1.2± 0.2 | | 0.7 ± 0.1 | | 0.9 ± 0.1 | | 0.5± 0.1 | | | Factor 1: F=0.47; p=0.49  Factor 2: F=1.33; p=0.25  Interaction F= 0.01; p=0.88 | | |  |
| Stretching in the open arms | 9.4± 0.9 | | 2.7 ± 0.8 | | 3.0 ± 0.6 | | 3.0± 0.6 | | | Factor 1: F=2.42; p=0.13  Factor 2: F=2.89; p=0.10  Interaction F= 2.89; p=0.10 | | |  |
| Stretching in the closed arms | 0.3± 0.1 | | 3.0 ± 0.3 | | 1.6 ± 0.3 | | 1.8± 0.3 | | | Factor 1: F=0.14; p=0.71  Factor 2: F=1.80; p=0.19  Interaction F= 0.23; p=0.63 | | |  |
| Time spent in stretching – open arms | 3.1± 0.3 | | 1.0 ± 0.3 | | 0.8 ± 0.1 | | 0.9± 0.2 | | | Factor 1: F=2.93; p=0.09  Factor 2: F=2.12; p=0.15  Interaction F= 2.89; p=0.10 | | |  |
| Time spent in stretching – open arms extremities | 1.3± 0.2 | | 0.5 ± 0.2 | | 0.3 ± 0.1 | | 0.3± 0.0 | | | Factor 1: F=2.48; p=0.12  Factor 2: F=0.89; p=0.35  Interaction F= 0.73; p=0.39 | | |  |
